# *Mycobacterium tuberculosis* Rv3463 induces mycobactericidal activity in macrophages by enhancing phagolysosomal fusion and exhibits therapeutic potential

**DOI:** 10.1038/s41598-019-38982-0

**Published:** 2019-03-12

**Authors:** Hye-Soo Park, Yong Woo Back, Ki-Won Shin, Hyun Shik Bae, Kang-In Lee, Han-Gyu Choi, Seunga Choi, Hwang-Ho Lee, Chul Hee Choi, Jeong-Kyu Park, Hwa-Jung Kim

**Affiliations:** 1 Department of Microbiology and Department of Medical Science, College of Medicine, Chungnam National University, Daejeon, Republic of Korea; 2 Department of Microbiology and Immunology, Chonbuk National University Medical School, Jeonju, Republic of Korea

## Abstract

Macrophages are responsible for innate and adaptive immune response activation necessary for eliminating infections. Optimal activation of macrophages to phagocytize *Mycobacterium tuberculosis* is critical in anti-mycobacterial defense. Here, we identified a novel Rv3463 hypothetical protein that induces macrophage activation in Mtb culture filtrate. Recombinant Rv3463 activated mouse bone marrow-derived macrophages to induce the expression of surface molecules and secrete pro-inflammatory cytokines via the TLR2 and TLR4 pathways. Mitogen activated protein kinase, phospatidylinositol-4,5-bisphosphate 3-kinases, and the NF-κB signaling pathways are involved in Rv3463-mediated macrophage activation. Furthermore, Rv3463 induced bactericidal effects in Mtb-infected macrophages through phagosome maturation and phagolysosomal fusion enhanced by phospatidylinositol-4,5-bisphosphate 3-kinases and Ca^2+^ signaling pathways and exhibited therapeutic effects in a short-term Mtb-infection mouse model. Overexpression of Rv3463 in *M. smegmatis* caused rapid clearance of bacteria in macrophages and mice. Our study suggests that Rv3463 is a promising target for the development of post-exposure tuberculosis vaccines or adjunct immune-therapy.

## Introduction

*Mycobacterium tuberculosis* (Mtb) is one of the most infectious intracellular pathogens, infecting one-third of the population in the world. Individuals possessing latent Mtb have a 10% lifetime risk of developing reactivation tuberculosis (TB), and this risk is markedly increased in immunosuppressed patients^1,2^. However, the only available vaccine, *M. bovis* BCG, is not fully effective for protection against adult pulmonary TB as well as reactivation of latent TB^3^. These issues present an urgent need for a better understanding of factors related to mycobacterial survival, which could lead to the development of novel strategies to eradicate Mtb from infected hosts.

Mtb can consistently survive and grow under a hostile environment such as that of macrophages^4^. Alternatively, the host cells that interact with Mtb initiate diverse protective responses to control bacterial growth. The overall anti-mycobacterial defense mechanism has been known, whereby macrophages phagocytose Mtb and secrete pro-inflammatory cytokines or chemokines, and the T cells activated by dendritic cells that capture Mtb stimulate the macrophages to kill the bacteria within the phagosomes. Many studies have clearly demonstrated the essential role of T cells in controlling Mtb growth, but adaptive T cell responses do not completely eradicate Mtb, resulting in latent TB^5^. Therefore, understanding the functional interaction between immune cells and Mtb or its components is necessary for achieving a bactericidal immune response and developing novel therapeutic strategies.

Macrophages are the main effector cells that eliminate mycobacteria. After phagocytosis, macrophages must undergo phagosome maturation, and subsequently, mature phagosomes interact with endosomes and lysosomes, leading to the acidification of phagolysosomes, which results in the degradation and clearance of Mtb^6,7^. At the same time, processes such as Mitogen- activated protein kinases (MAPKs) and calcium signaling are initiated; these processes play important roles in the bactericidal response to infected cells^8^. However, pathogenic mycobacterial species have developed strategies to interfere with phagosome maturation such as endosomal trafficking, acidification of the phagosome, and fusion with the lysosome^9^. Mtb also inhibits a rise in cytosolic Ca^2+^, which is critical for PI3P-dependent phagolysosome biogenesis^10^. Several mycobacterial factors including SapM^11^, PtpA^12^ and lipoarabinomannan (LAM)^13^ have been previously reported to modulate the phagosome maturation process. Therefore, optimal activation of macrophages, which play an important role in the effector phase of the immune response, is critical in anti-mycobacterial defense. Mtb contains diverse proteins that activate macrophages to induce secretion of anti-inflammatory^14^ or pro-inflammatory cytokines^15^. However, little is known about the protective role and bactericidal mechanisms of these macrophage-activating proteins.

Although candidate TB vaccines have focused on T-cell stimulating antigens, we postulate that proteins that induce the bactericidal activity in macrophages can be ideal vaccine targets, particularly for the development of a post-infectious vaccine. Recently, we reported that Rv2882c protein induce macrophage activation and exhibit potential for use as a vaccine such as the BCG booster^15^. In this study, we identified and characterized the immunoreactivity of a novel macrophage-activating protein from Mtb culture filtrate proteins (CFPs) by multidimensional fractionation. Rv3463, a newly identified hypothetical protein, activated macrophages to induce mycobactericidal activity that was strongly associated with rise in phospatidylinositol-4,5-bisphosphate 3-kinase (PI3K) and an intracellular Ca^2+^. In addition, Rv3463 expression in *M. smegmatis* caused rapid clearance in macrophages and mice. Moreover, Rv3463 exhibited therapeutic potential in a Mtb-infected mouse model. These findings suggest that Rv3463 is a promising candidate for TB immunotherapy.

## Results

### Identification and preparation of Rv3463 protein from Mtb culture filtrates

**Figure 1:**
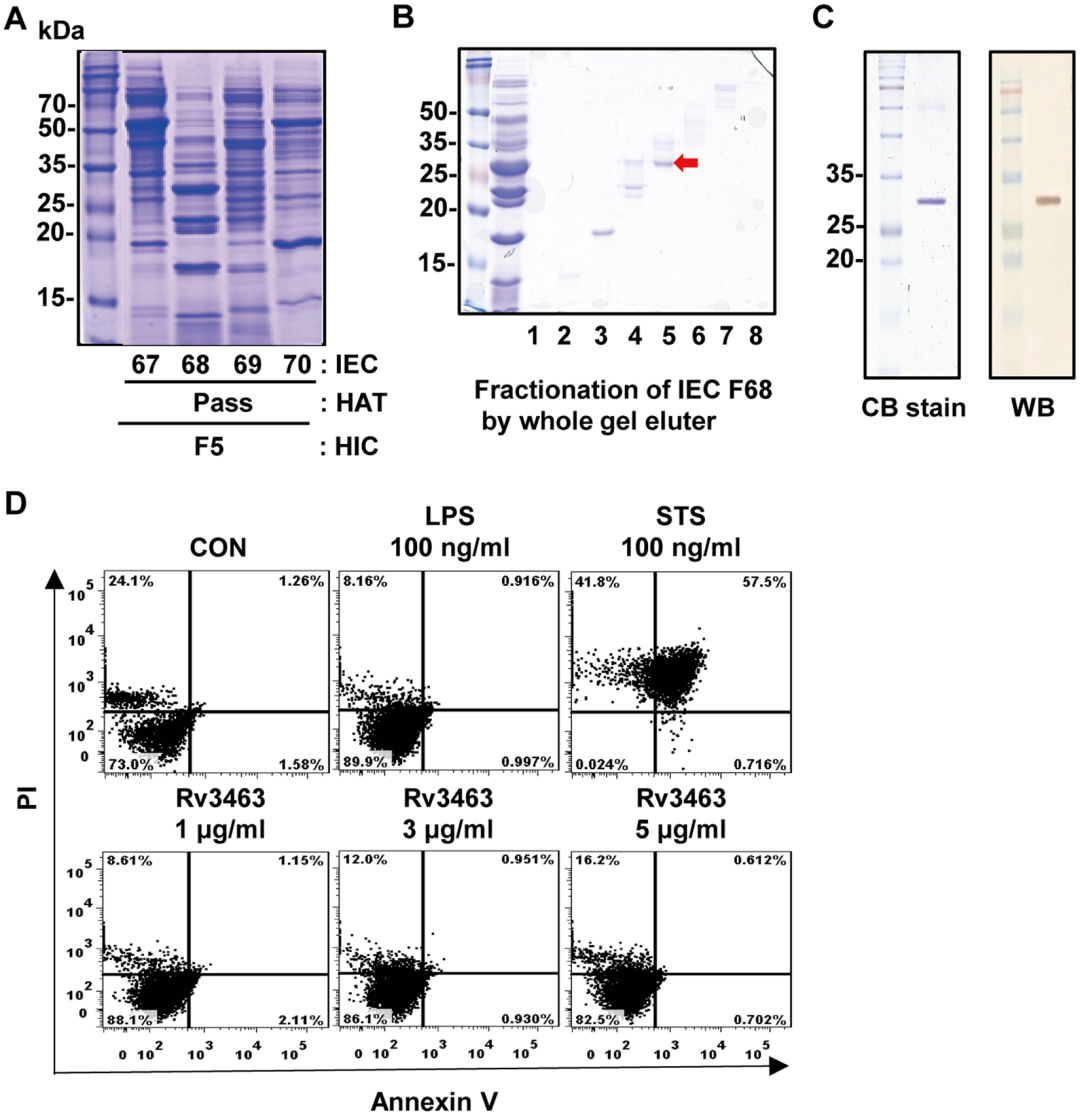
Identification and preparation of Rv3463 protein. (**A**) Ammonium sulfate precipitate of *Mycobacterium tuberculosis* (Mtb) culture filtrate was fractionated with hydrophobic interaction chromatography (HIC) using Phenyl Sepharose. The primary fractions were sequentially fractionated by hydroxylapatite chromatography (HAT) and ion-exchange chromatography (IEC). (**B**) Fractions of interest were further separated by mini-whole gel eluter. (**C**) Recombinant Rv3463 purified from *E. coli* extracts was subjected to SDS-PAGE and western blot (WB) analysis using a mouse anti-His antibody. All proteins were analyzed by SDS-PAGE with Coomassie blue staining. (**D**) The cytotoxic effects of Rv3463 were analyzed by flow cytometry. BMDMs were stimulated with Rv3463, LPS or staurosporine (STS) for 24 h, and then stained with Annexin V and PI. The percentage of cells that are positive in each quadrant is indicated. The results are representative of three experiments.

Mtb culture filtrates were fractionated by a multistep chromatography as described previously^15^. In brief, the 80% ammonium sulfate precipitates of the culture filtrates were primarily separated into seven fractions by hydrophobic interaction chromatography. Each initial fraction was further fractionated by hydroxylapatite chromatography (HAT) and ion-exchange chromatography (IEC). Among the final fractions, IEC fraction number 68 from HAT pass fraction of the initial fraction 5 showed strong reactivity to secreted pro-inflammatory cytokines in macrophages as well as multiple bands on a SDS-PAGE gel (Fig. 1A). Therefore, this fraction was further separated by a miniwhole gel eluter. The major band in fraction number 5 with strong reactivity (marked by the arrow in Fig. 1B) was identified as Rv3463 by liquid chromatography electrospray ionization-tandem mass spectrometry (LC-ESI/MS). A recombinant Rv3463 protein was purified from *Escherichia coli*. Purified recombinant Rv3463 appeared as a 29 kDa major band and reacted with an anti-His antibody (Fig. 1C). The endotoxin content was measured by limulus amoebocyte lysate (LAL) assay, and only the protein lots with very low endotoxin content (<0.2 EU/ml) were used in subsequent experiments. As shown in Fig. 1D, there were no cytotoxic responses in bone marrow-derived macrophages (BMDMs) treated with 5 μg/ml Rv3463 for 24 h.

### Rv3463 activates macrophages to express surface molecules and secrete pro-inflammatory cytokines

**Figure 2:**
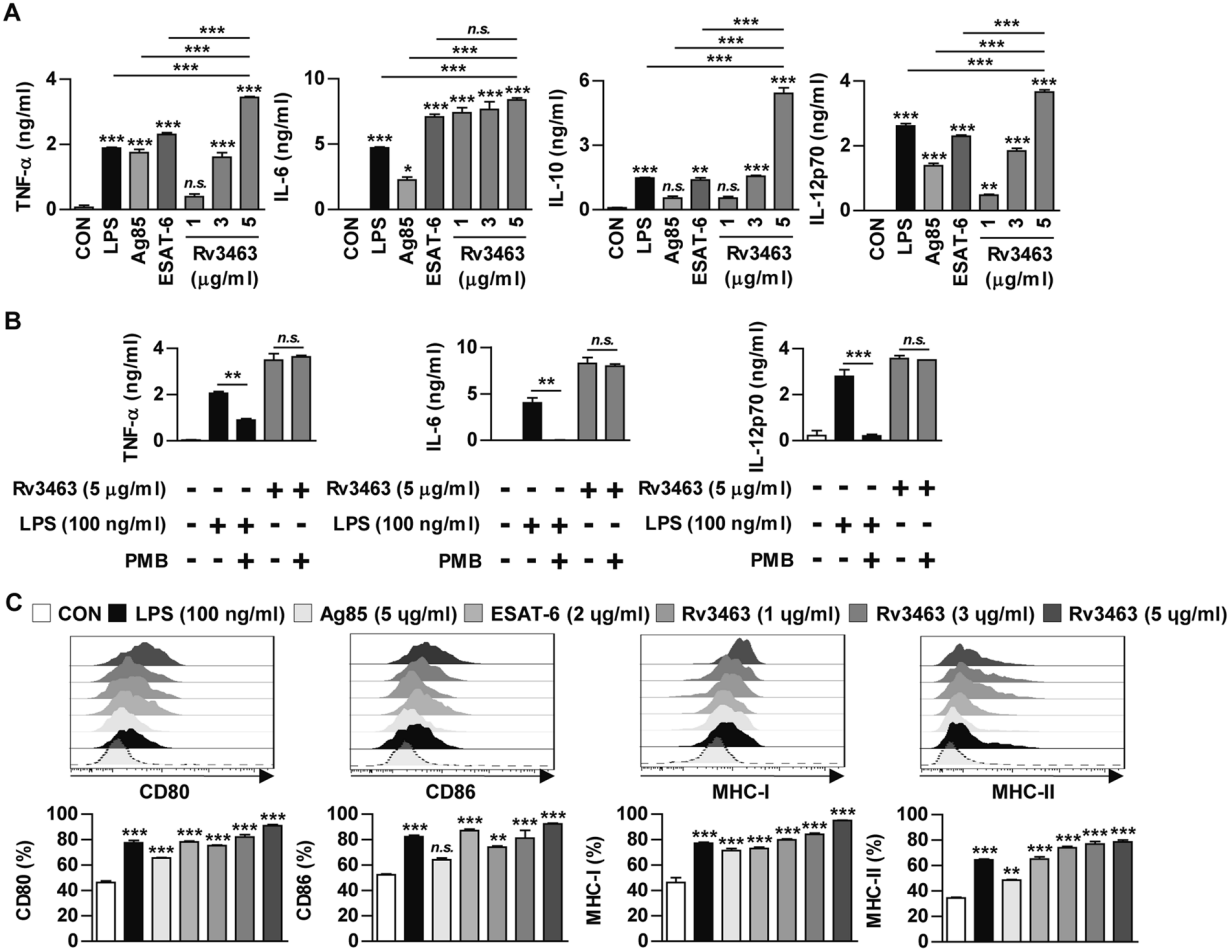
Rv3463 induces bone marrow-derived macrophages (BMDMs) activation. BMDMs were stimulated with Rv3463 (1, 3 or 5 μg/ml), lipopolysaccharide (LPS, 100 ng/ml), Ag85 (5 μg/ml), or ESAT-6 (2 μg/ml) for 24 h. (**A**) The cytokines from the culture supernatants were measured by ELISA. All data are expressed as mean ± SD (*n* = 3). (**C**) BMDMs stimulated with each antigen were also analyzed for the expression of surface markers by two-color flow cytometry. The cells were gated to exclude F4/80^+^ cells. BMDMs were stained with anti-CD80, anti-CD86, anti-MHC class I or anti-MHC class II antibodies. The histograms are representative of five experiments. Bar graphs show the percentage (mean ± SD of five experiments) for each surface molecule on F4/80^+^ cells. (**B**) BMDMs were incubated with LPS (100 ng/ml) or Rv3463 (5 μg/ml) with or without pretreatment of polymyxin B (PMB) for 1 h. After 24 h, TNF-α, IL-6 and IL-12p70 production was analyzed by ELISA from culture supernatants. All data are expressed as mean ± SD (*n* = 3). *p < 0.05, **p < 0.01 and ***p < 0.001 for treatment compared to untreated controls (CON) or for difference between treatment data. *n.s*., no significant difference.

Next, the effect of recombinant Rv3463 on macrophage activation was tested. The cytokine levels were determined in the culture supernatant of BMDMs treated with Rv3463 at 1, 3, and 5 μg/ml for 24 h. Lipopolysaccharide (LPS) (100 ng/ml) was used as a positive control. Recombinant Ag85 (5 μg/ml) and ESAT-6 (2 μg/ml), which are immunodominant Mtb antigens, were also used as a comparable mycobacterial antigen. Rv3463 induced significant production of TNF-α, IL-6, IL-12p70, and IL-10 in a dose-dependent manner when compared to that observed in untreated cells (Fig. 2A). BMDMs treated with Rv3463 at a concentration of 5 μg/ml secreted a significant amount of TNF-α and IL-12p70 when compared to those treated with Ag85, ESAT-6, or LPS, while also producing a significantly higher amount of IL-10 compared to other antigens. We further confirmed that Rv3463-mediated cytokine production was not due to LPS contamination (Fig. 2B), although LPS was removed from the purified Rv3463 protein. Next, to investigate whether Rv3463 could affect the antigen presentation of macrophages, we determined the expression levels of MHC (class I and II) and co-stimulatory molecules in macrophages by flow cytometry. As demonstrated in Fig. 2C, BMDMs treated with Rv3463 significantly enhanced the expression of CD80 and CD86 surface molecules as well as MHC class I and II molecules in a dose-dependent manner when compared to the untreated control, and Rv3463 activity in the expression of these surface molecules was comparable to other antigens. These data suggest that Rv3463 can effectively induce macrophage activation.

### Rv3463 induces macrophage activation through the TLR2 and TLR4 pathways and its down-stream signaling molecules MAPKs, PI3K and NF-κB

**Figure 3:**
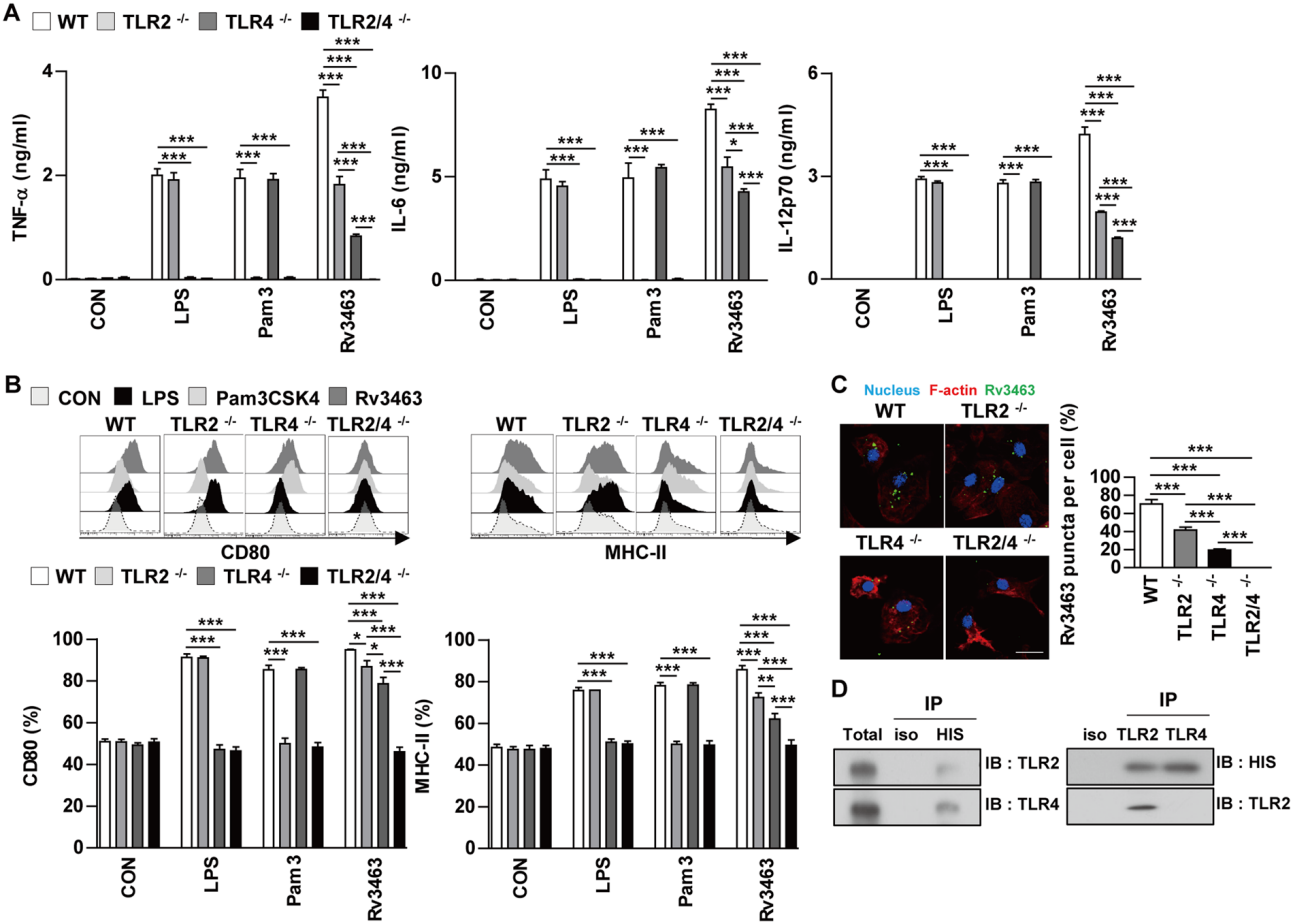
Rv3463 induces macrophage activation via TLR2 and TLR4. Bone marrow-derived macrophages (BMDMs) derived from wild-type (WT), TLR2^−/−^, TLR4^−/−^, and TLR2/4^−/−^ mice were treated with Rv3463 (5 μg/ml), lipopolysaccharide (LPS, 100 ng/ml), or Pam3CSK4 (100 ng/ml) for 24 h. (**A**) The production of TNF-α, IL-6, and IL-12p70 in the culture supernatants was determined by ELISA. All data are expressed as mean ± SD (*n* = 3). (**B**) Expression of CD80 and MHC class II molecules on BMDMs stimulated with each antigen was determined by staining and flow cytometry. The bar graphs show the mean percentage ± SEM of each surface molecule on F4/80^+^ cells across three independent experiments. *p < 0.05, **p < 0.01 and ***p < 0.001 for treatment values in BMDMs from TLR2^−/−^, TLR4^−/−^ or TLR2/4^−/−^ mice compared to those of Rv3463-, LPS- or Pam3CSK4-treated BMDMs from WT mice. (**C**) Fluorescence intensities of anti-Rv3463 bound to the surface of BMDMs. BMDMs derived from WT, TLR2^−/−^, TLR4^−/−^, and TLR2^−/−^ mice were treated with Rv3463 (5 μg/ml) for 1 h, fixed, and then stained with DAPI (blue) and Alexa488-conjugated anti-Rv3463 antibody. Representative images out of three independent experiments are shown. Scale bar, 10 μm. ***p < 0.001 for difference between BMDMs from each mice. (**D**) The cell lysates from BMDMs treated with Rv3463 for 6 h were used for immunoprecipitation with anti-mouse IgG and anti-His (upper), or anti-TRL2 and TLR4 antibodies (lower). Thereafter, proteins were detected using immunoblotting with anti-His, anti-TLR2 or anti-TLR4 antibodies. The total is shown as the mean total cell lysates (input).

Mtb and its components activate macrophages or dendritic cells through interactions with the TLR signaling pathway^15,16^. We determined the involvement of TLR signaling in Rv3463-mediated macrophage activation by using the BMDMs prepared from C57BL/6J wild-type (WT), TLR2^−/−^, TLR4^−/−^, and TLR2/4^−/−^ mice. Rv3463-induced cytokine production and expression of surface molecules including CD80 and MHC class II were significantly depressed in BMDMs from TLR2^−/−^, and TLR4^−/−^ mice compared to BMDMs from WT mice (Fig. 3A,B). As expected, LPS and Pam3CSK4 activities were significantly decreased in BMDMs from TLR4^−/−^ and TLR2^−/−^ mice, respectively. In particular, cytokine production and expression of the surface molecules induced by Rv3463 were significantly suppressed in BMDMs from TLR2/4^−/−^ mice (Fig. 3A,B) when compared to those from the TLR2^−/−^ or TLR4^−/−^ mice. Next, we used confocal microscopy to investigate whether Rv3463 could directly interact with TLR2 and TLR4. As shown in Fig. 3C, Rv3463 interacted with the surface of BMDMs from WT mice; however, binding to BMDMs from TLR2^−/−^ and TLR4^−/−^ mice was prominently decreased. BMDMs from TLR2/4^−/−^ mice did not bind to Rv3463 at all. Immunoprecipitation analysis using anti-His or anti-TLR2 and -TLR4 antibodies revealed that Rv3463 mainly interacts with TLR4 while only slightly interacting with TLR2 (Fig. 3D). These results suggest that Rv3463 induces macrophage activation through the TLR2 and TLR4 pathways.

MAPKs, PI3K, and NF-κB are down-stream signaling molecules of TLR signaling pathway, and are crucial for regulating the macrophages activation^17,18^. Therefore, we investigated the mechanism involved in Rv3463-mediated signaling events responsible for activating macrophages. BMDMs were stimulated with Rv3463 at a concentration of 5 μg/ml for the indicated time points. As expected, Rv3463 triggered the phosphorylation of ERK1/2, p38, and JNK as well as the, phosphorylation and degradation of IκB-α (Supplementary Fig. 1A). Significant translocation of p65 from the cytosol to the nucleus was also observed (Supplementary Fig. 1B). In addition, phosphorylation of PI3K and Akt was increased in Rv3463-treated BMDMs. Next, we clarified the role of MAPKs, PI3K, and NF-κB in Rv3463-mediated macrophage activation with specific inhibitors. Rv3463-induced expressions of CD80 and CD86 (Supplementary Fig. 1C), and production of TNF-α, IL-10, and IL-12p70 production (Supplementary Fig. 1D) were attenuated by pretreatment with pharmacological inhibitors. Our data demonstrated that the MAPKs, PI3K, and NF-κB signaling pathways were essential for Rv3463-mediated macrophage activation.

### Rv3463 maintains the active state of Mtb-infected macrophages

**Figure 4:**
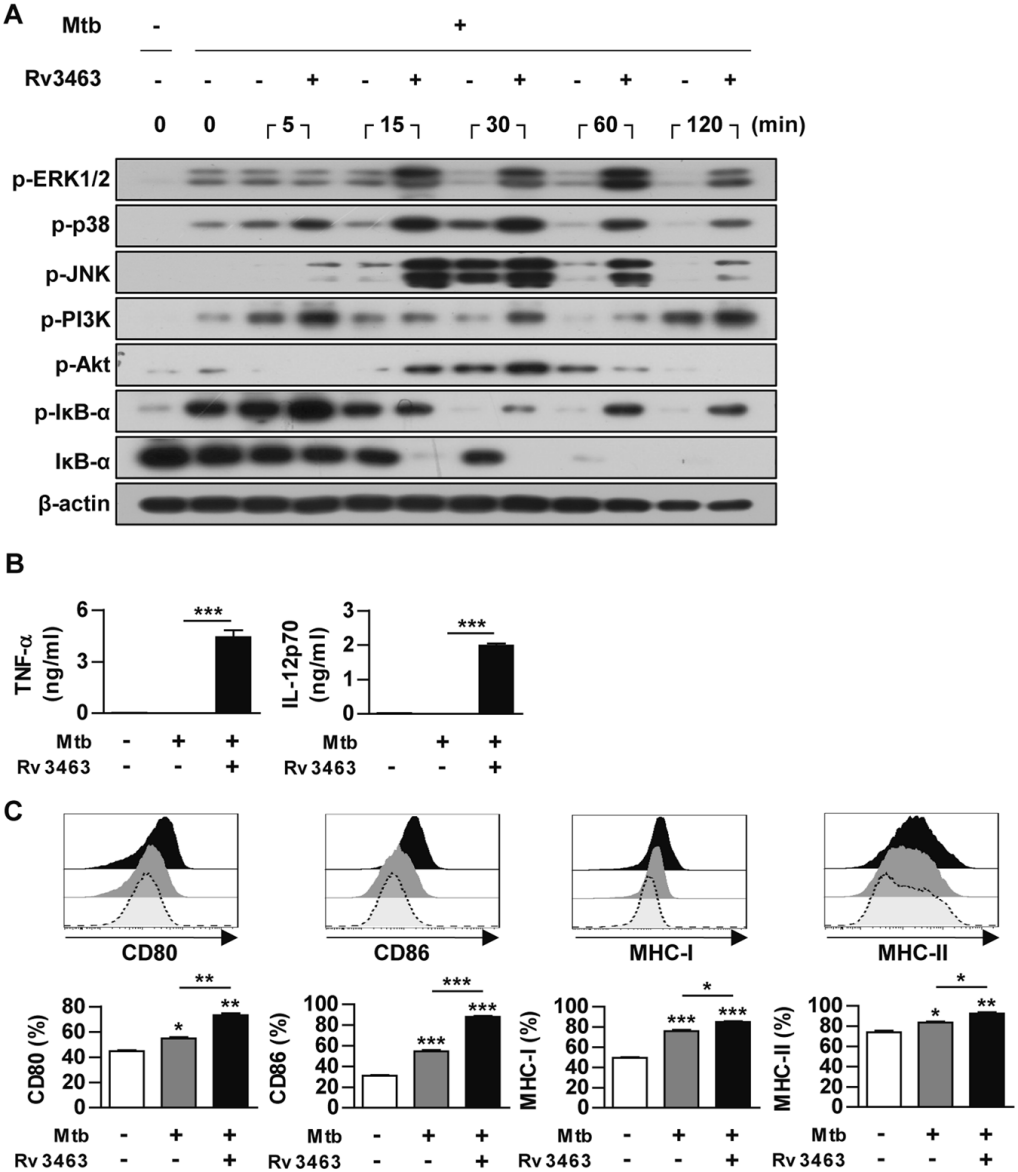
Rv3463 maintains the active state of *Mycobacterium tuberculosis* (Mtb)-infected macrophages. (**A**) Bone marrow-derived macrophages (BMDMs) were infected with Mtb at a multiplicity of infection (MOI) of 1 for 2 h, stimulated with or without 5 μg/ml Rv3463 for the times indicated. The cells were then lysed, and total cell lysates were separated by SDS-PAGE, followed by immunoblot analysis using antibodies against phospho-ERK1/2, phospho-p38, phospho-JNK, phospho-PI3K, phospho-Akt, phospho-IκB-α, IκB-α, and β-actin. The image is representative of three experiments showing similar results. (**B,C**) BMDMs infected with Mtb for 4 h were incubated with or without 5 μg/ml Rv3463 for 72 h. TNF-α and IL-12p70 in the culture supernatants were measured by ELISA. Data shown are mean ± SD (*n* = 3) (**B**). The expressions of surface molecules on the BMDMs were analyzed by flow cytometry. Histograms are representative of three experiments. Bar graphs show the percentage (mean ± SD of three experiments) for each surface molecule on F4/80^+^ cells (**C**). *p < 0.05, **p < 0.01 and ***p < 0.001 for Rv3463-treatment compared to infection only controls.

Virulent Mtb modulates the host protective response to increase their survival. Therefore, we investigated the stimulating effects of Rv3463 in Mtb-infected macrophages. Rv3463 continuously enhanced the phosphorylation of MAPKs, including p38, ERK1/2, JNK, PI3K, and Akt, and the phosphorylation and degradation of IκB-α (Fig. 4A). TNF-α and IL-12p70 production (Fig. 4B) and expression of surface molecules (Fig. 4C) in the Mtb-infected macrophages were significantly enhanced by Rv3463 treatment. These results suggested that Rv3463 count Mtb-mediated down regulation of macrophage activation.

### Rv3463 inhibits the growth of intracellular Mtb by enhancing phagolysosomal fusion in Mtb-infected macrophages

**Figure 5:**
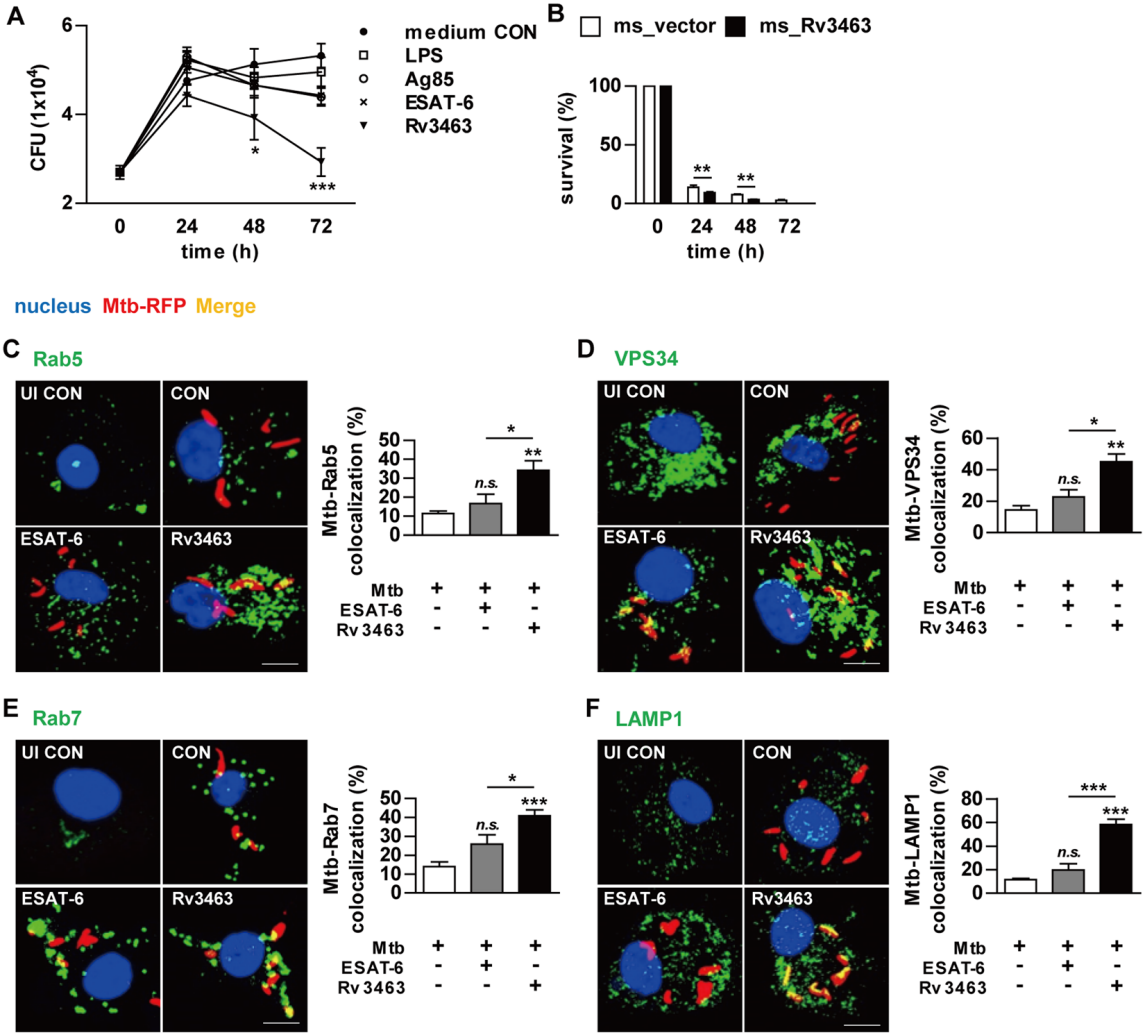
Rv3463 inhibits the growth of intracellular Mtb through induction of phagolysosomal fusion in Mtb-infected macrophages. (**A**) Bone marrow-derived macrophages (BMDMs) were infected with Mtb at a multiplicity of infection (MOI) of 1 for 4 h, and then further treated with amikacin to kill extracellular bacteria for 2 h, washed three times, and incubated with or without 5 μg/ml Rv3463, 100 ng/ml LPS, 5 μg/ml Ag85, or 2 μg/ml ESAT-6 for 72 h. Intracellular Mtb growth was determined by plating the cell lysates on 7H10 agar for 0 to 72 h. (**B**) BMDMs were infected with *M. smegmatis* expressing Rv3463 (ms_Rv3463) and vector control strain (ms_vector) (MOI = 10) for 4 h, and then further treated with gentamycin for 2 h and washed three times. At 24, 48, and 72 h post-infection, intracellular bacterial growth was determined. Similar results were obtained for three independent experiments. **p < 0.01 for ms_Rv3463 compared to vector control strain. (**C–F**) BMDMs were infected with Mtb-RFP (MOI = 1) for 4 h, washed, incubated with or without 5 μg/ml Rv3463 or 2 μg/ml ESAT-6 for 72 h, fixed with 4% paraformaldehyde, and immunolabeled with anti-Rab5 (**C**), anti-VPS34 (**D**), anti-Rab7 (**E**), or anti-LAMP1 (**F**) antibodies, followed by Alexa 488-conjugated goat anti-rabbit IgG or anti-rat IgG (green). Cells were stained with DAPI to visualize the nuclei (blue). The localizations of the target molecules were analyzed by laser-scanning confocal microscopy. Scale bar, 10 μm. Bar graphs show the quantification of confocal data for the colocalization of Mtb and molecules detected. Values are mean ± SD of 50–100 cells per each experiment (*n* = 3). ***p < 0.001 for treatment compared to infection only controls (CON) or for difference between treatment data. *n.s*., no significant difference.

We next investigated whether Rv3463-activated macrophages could affect intracellular Mtb growth. BMDMs were infected with Mtb for 4 h and then stimulated with the antigens. To screen the effects of Rv3463 on the bactericidal activity of macrophages, BMDMs were infected with RFP-expressing Mtb and then stimulated with the antigens. Flow cytometric analysis demonstrated that the fluorescence intensity was most significantly reduced in BMDMs stimulated with Rv3463 compared to other antigens (Supplementary Fig. 2). Determination of Mtb growth on 7H10 agar revealed that Mtb growth in Rv3463-stimulated BMDMs was significantly limited in a time-dependent manner when compared to those treated with LPS, Ag85, or ESAT-6 (Fig. 5A). At 72 h post-infection, the production of IL-12p70 and TNF-α was significantly enhanced in BMDMs treated with Rv3463 when compared to non-treated infected cells or cells stimulated with other antigens (Supplementary Fig. 3). To confirm whether TLR signaling played a role in Rv3463-stimulated cells, we assessed the growth of Mtb within BMDMs from WT, TLR2^−/−^, and TLR4^−/−^ mice. As expected, Rv3463-mediated restriction of the intracellular growth of Mtb was observed in BMDMs from WT mice but not in the BMDMs from all knockout mice (Supplementary Fig. 4A). To further confirm the role of Rv3463 under condition of whole bacteria, we constructed a strain of *M. smegmatis* overexpressing Rv3463. Rv3463 expression in the recombinant *M. smegmatis* strain was confirmed by western blot analysis (Supplementary Fig. 5A), and there was no significant difference in the growth rates between the recombinant and vector control strains in the culture media (Supplementary Fig. 5B). We further tested the effect of Rv3463 on the viability of *M. smegmatis* by alamarBlue assay^19^. The bacterial viability was similar between ms_vector and ms_Rv3463 (Supplementary Fig. 1C), suggesting that Rv3463 does not affect mycobacterial growth. However, the survival rate of *M. smegmatis* expressing Rv3463 in BMDMs was significantly lower than that of the vector control strain (Fig. 5B).

The diverse host defense mechanisms to control mycobacteria in macrophages involve the induction of apoptosis, recruiting of help from T cells via antigen presentation, fusion of the phagosome and lysosome, and stimulation of bactericidal pathways such as MAPKs, IFN-γ, and Ca^2+^ signaling. Among them, phagosome maturation is one of the most important mechanisms for the elimination of bacteria in macrophages^4,20^. Therefore, we investigated the effects of Rv3463 on phagolysosome biogenesis during Mtb infection. For this, BMDMs infected with RFP-expressing Mtb were cultured in the presence of Rv3463 or ESAT-6, and then the expression of Rab5, VPS34, Rab7, and LAMP1 was assessed by confocal microscopy. Rv3463 significantly increased the co-localization of the early endosome marker Rab5 or VPS34 to the Mtb phagosome when compared to the control or ESAT-6-treated cells (Fig. 5C,D). Furthermore, localization of the late endosomal maker Rab7 to the Mtb-containing phagosome was significantly increased by Rv3463 treatment compared to that in control or ESAT-6-treated cells (Fig. 5E). In particular, Rv3463 treatment significantly facilitated the acquisition of the lysosome marker LAMP1 to Mtb phagosome compared to that by ESAT-6 treatment (Fig. 5F). However, Rv3463-induced LAMP1 localization to Mtb phagosome was not observed in BMDMs from TLR2^−/−^ and TLR4^−/−^ mice (Supplementary Fig. 4B). Localization of Rab5, VPS34, Rab7, and LAMP1 into phagosomes containing *M. smegmatis* expressing Rv3463 was prominently increased when compared to the vector control strain (Supplementary Fig. 6).

Autophagy is an important feature in the elimination of Mtb^21,22^. Therefore, we assessed whether Rv3463 could induce autophagosome accumulation by detection of microtubule-associated protein light-chain 3 (LC3) with immunofluorescence staining. Formation of endogenous LC3 punctate was increased in macrophages infected with Mtb or treated with rapamycin (Rapa), an autophagy inducer that blocks Mtor; however, the LC3 punctate in Mtb-infected BMDMs was not enhanced by Rv3463 (Supplementary Fig. 7A). Similar results were observed by western blot analysis for the detection of endogenous LC3-II protein levels (Supplementary Fig. 7B). These results suggest that Rv3463-mediated inhibition of Mtb growth in macrophages was strongly associated with enhancing phagolysosomal fusion, and was thus not associated with autophagy.

### PI3K inhibition disrupts Rv3463-induced antimicrobial effects, pro-inflammatory cytokine production, and phagolysosome fusion

**Figure 6:**
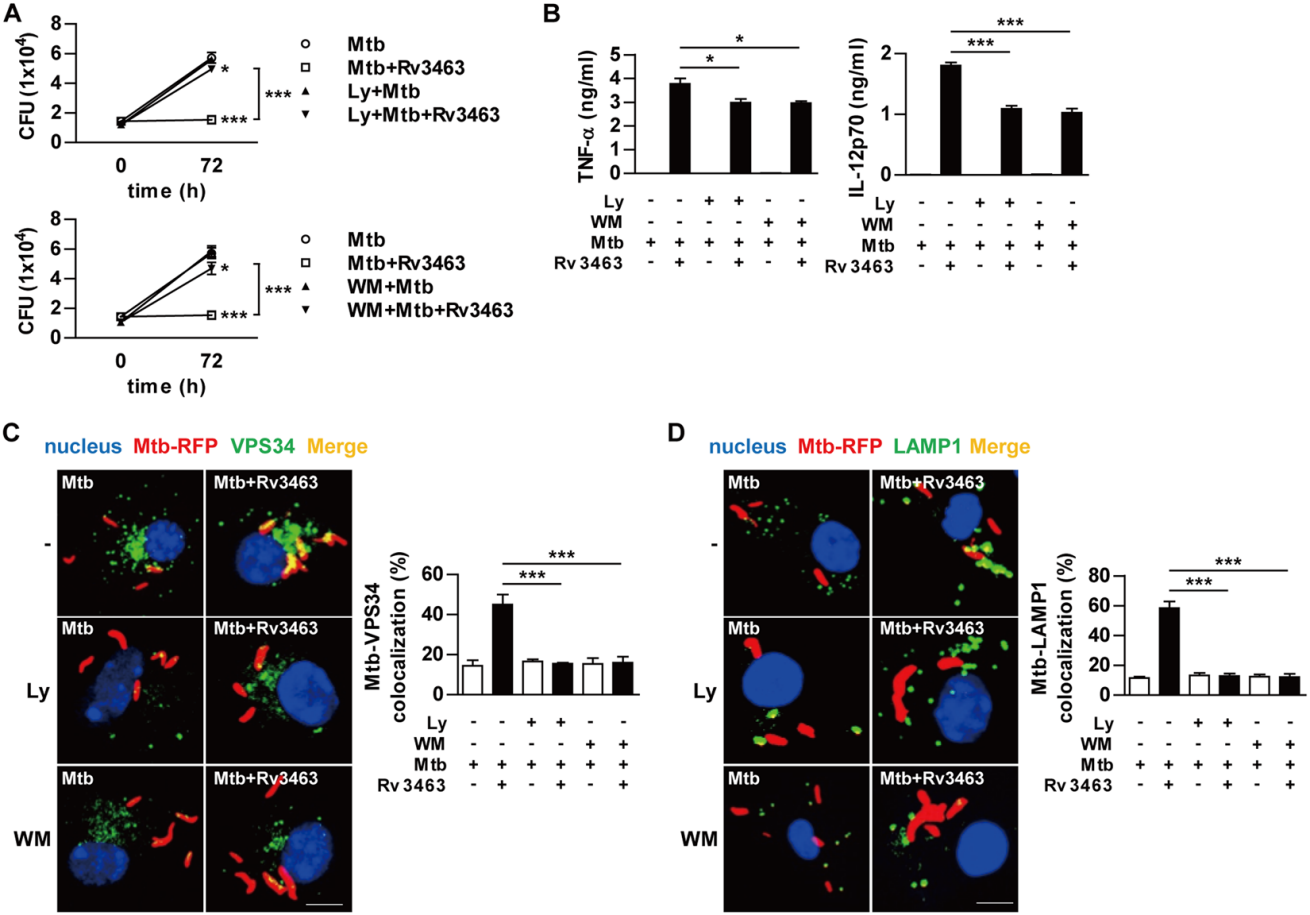
PI3K inhibition interferes the Rv3463-mediated activities. Bone marrow-derived macrophages (BMDMs) pretreated with pharmacological inhibitors of PI3K (20 μM LY2940029 [Ly] or 200 nM Wortmannin [WM]) for 1 h were infected with Mtb (**A,B**) or Mtb-RFP (**C,D**) at a multiplicity of infection of 1 for 4 h, treated with amikacin, washed, and incubated with or without 5 μg/ml Rv3463 for 72 h. (**A**) Intracellular Mtb growth in the cells was determined at 0 and 72 h. *p < 0.05 and ***p < 0.001 for treatment compared to infection only control. (B) TNF-α or IL-12p70 production in culture supernatants were measured by ELISA. (**C,D**) Colocalization of VPS34 or Lamp1 molecules (green) with Mtb (red) in the treated BMDMs were analyzed by laser-scanning confocal microscopy. The cells were stained with DAPI to visualize the nuclei (blue). Scale bar, 10 μm. Bar graphs show the quantification of VPS34 or Lamp1 colocalization with the Mtb phagosome. Values are mean ± SD of 50–100 cells per each experiment (*n* = 3). *p < 0.05, **p < 0.01 and ***p < 0.001 for inhibitor treatment compared to the Rv3463-treated cells.

PI3K is activated by many different signaling molecules and is crucial for cell growth and survival. In particular, PI3K activity is essential for phagosome maturation^23,24^. Because it catalyzes the production of PI3P, which is a phagosomal membrane tag protein for progression through the phagolysosome biogenesis pathway. We confirmed that the localization of VPS34, a type III PI3K, into Mtb-containing phagosomes was enhanced by Rv3463 treatment (Fig. 5D). Therefore, we hypothesized that PI3K plays a critical role in Rv3463-mediated Mtb growth inhibition. Western blot analysis confirmed that PI3K inhibitors blocked phosphorylation of PI3K as well as Akt which is downstream of PI3K (Supplementary Fig. 8). PI3K inhibitors alone (LY294002 or Wortmannin) did not affect the intracellular growth of Mtb, but significantly abrogate Rv3463-mediated Mtb growth inhibition (Fig. 6A). TNF-α and IL-12p70 production enhanced by Rv3463 in Mtb-infected macrophages was significantly suppressed by PI3K inhibitors (Fig. 6B). Furthermore, PI3K inhibitors suppressed Rv3463-mediated localization of VPS34 and LAMP1 to Mtb phagosomes (Fig. 6C,D). PI3K gene knock-down analysis showed the same results with PI3K inhibitors (Supplementary Fig. 9A–C). These results indicate that the Rv3463-mediated Mtb growth inhibition was dependent on PI3K.

### Rv3463 increases intracellular Ca^2+^ in Mtb-infected macrophages

**Figure 7:**
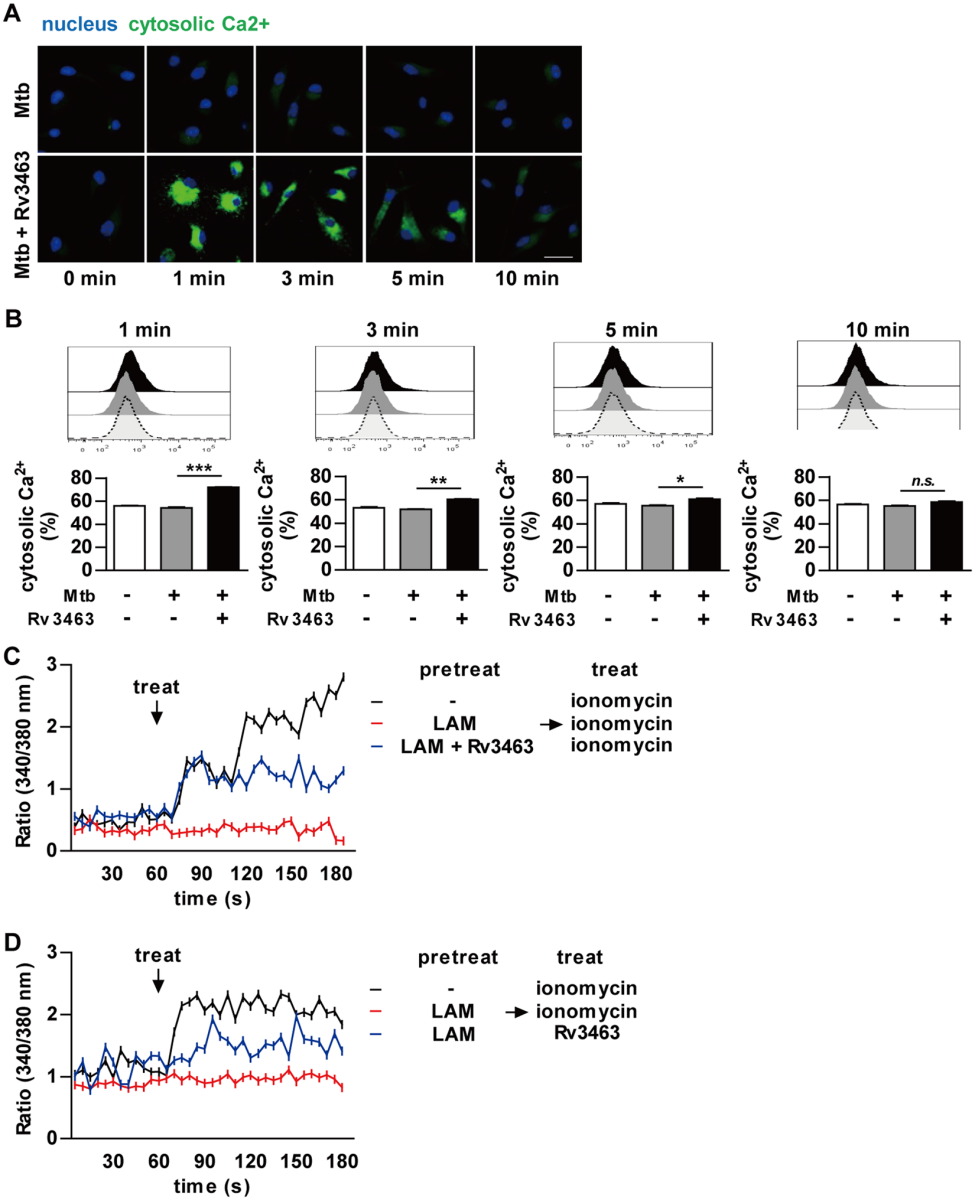
Rv3463 increases the intracellular Ca^2+^ in *Mycobacterium tuberculosis* (Mtb)-infected macrophages. (**A,B**) Bone marrow-derived macrophages (BMDMs) were infected with Mtb at a multiplicity of infection (MOI) 1 for 2 h, washed three times, and incubated with or without 5 μg/ml Rv3463 for the time indicated. The cells loaded with Flou-4/AM were analyzed by confocal microscopy (**A**) and by flow cytometry (**B**). Scale bar, 10 µm. (**C,D**) BMDMs were preincubated for 30 min with or without 20 μg/ml Mtb LAM or a mixture of LAM and Rv3463. Ratio (340/380 nm) kinetics at different time points after the addition of 500 nM ionomycin or 5 μg/ml Rv3463. The cells loaded with Fura-2/AM were analyzed by Nikon Eclipse microscopy. Mean ± SD, *n* = 30 cells. *p < 0.05, **p < 0.01 and ***p < 0.001 for treatment compared to infection only. *n.s*., no significant difference.

Previously, the importance of Ca^2+^ in phagolysosome biogenesis has been demonstrated^25^. Increases in intracellular Ca^2+^ after infection facilitate the recruitment of Rab5 to the mycobacterial phagosome, which subsequently leads to the recruitment of PI3K VPS34 to the phagosome, thereby increasing PI3P production in the phagosome membrane. Rv3463 induced the localization of VPS34 to the phagosome in macrophages. Therefore, we hypothesized that Rv3463-mediated bactericidal activity might be mediated by a rise in cytosolic Ca^2+^. We tested this possibility by determining the change of Ca^2+^ concentration in Mtb-infected BMDMs after Rv3463 treatment by using the fluo-4AM indicator. As shown in Fig. 7A, Rv3463 induced rapid increases in intracellular Ca^2+^ in Mtb-infected macrophages. Ca^2+^ concentration was the highest 1 min after Rv3463 treatment, and gradually decreased until 10 min after Rv3463 treatment (Fig. 7B). LAM blocks phagosomal maturation by inhibiting the rise in cytosolic Ca^2+^, which is an important Mtb survival mechanism in macrophages^26,27^. Therefore, we tested whether LAM could affect the Rv3463-mediated Ca^2+^ fluxes. As expected, preincubation of LAM inhibited the ionomycin-mediated Ca^2+^ increase in BMDMs, and interestingly, Ca^2+^ was increased in BMDMs co-pretreated with LAM and Rv3463 (Fig. 7C). LAM also did not interfere with the Rv3463-induced rise in Ca^2+^ under any condition, including pre-/post-treatment or simultaneous treatment with LAM (Fig. 7D and Supplementary Fig. 10). These results suggest that Rv3463 can induce bactericidal activity via Ca^2+^ fluxes and overwhelm the Mtb phagolysosomal fusion arrest mediated by LAM.

### Rv3463 exhibits therapeutic potential

**Figure 8:**
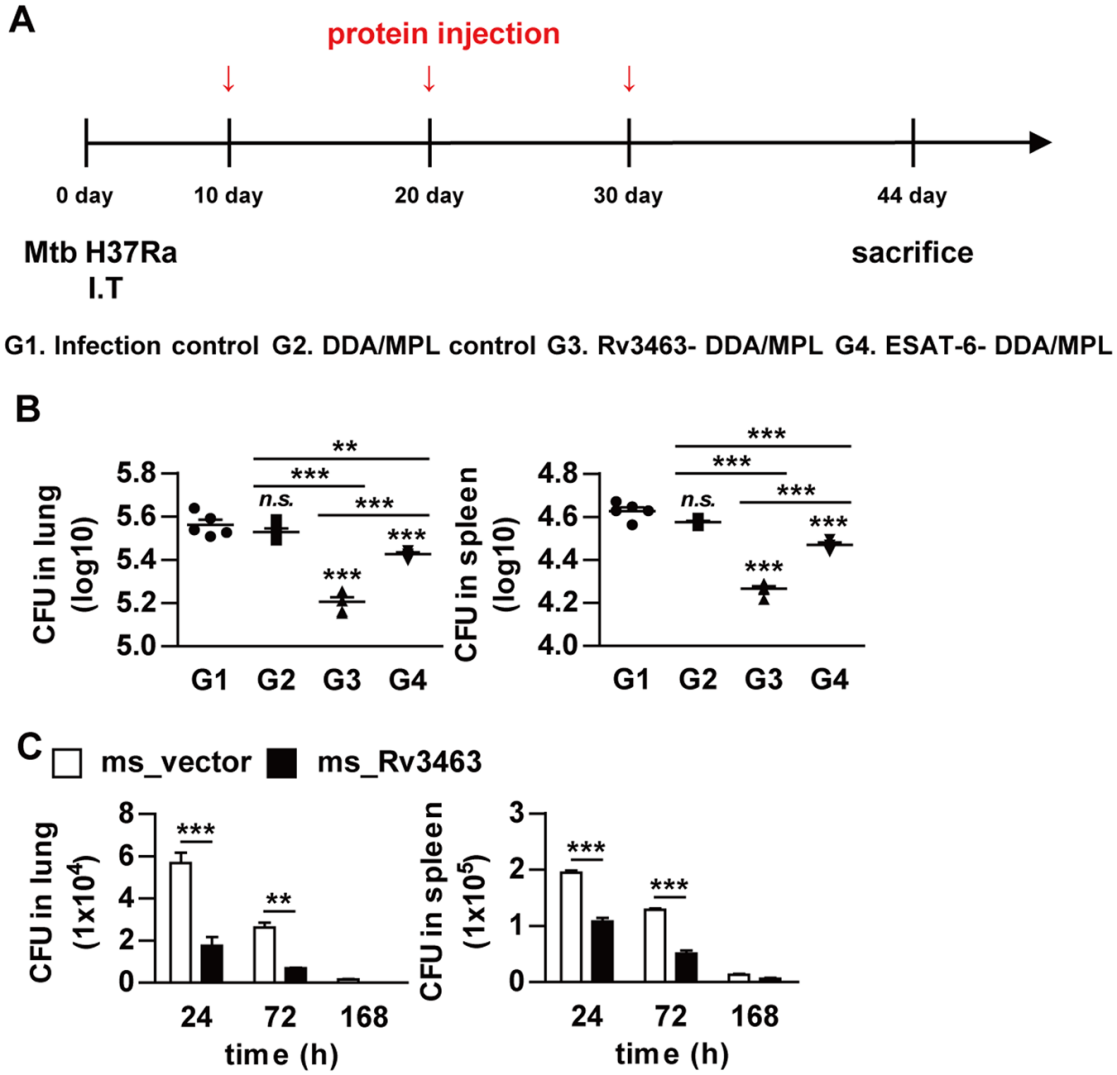
Therapeutic potential of Rv3463 *in vivo*. (**A**) A schematic diagram of the experimental design. C57BL/6 mice were infected intratracheally with 1 × 10^6^ CFU *Mycobacterium tuberculosis* (Mtb) per mouse, and then immunized three times with Rv3463 or ESAT-6. (**B**) Differences in the bacterial burden among mice groups treated with the protein-DDA/MPL, or the infection-only group, and those treated with the adjuvant control (DDA/MPL alone) at 4 weeks after the final immunization are shown in the lungs and spleens (*n* = 5 mice per group). **p < 0.01 and ***p < 0.001 compared to infection only group. *n.s*., no significant difference. (**C**) C57BL/6 mice infected intravenously with Rv3463-expressing *M. smegmatis* (ms_Rv3463) and vector controls strain (ms_vector) (1 × 10^6^ CFU per mouse) were sacrificed at the indicated time points (*n* = 3 per group at each time point) and then CFUs were determined in the lungs and spleens. **p < 0.01 and ***p < 0.001 compared vector controls.

Currently, there are no reliable protein candidates to be used as post-exposure or therapeutic vaccines for TB. Optimal induction of bactericidal activity in macrophages is critical to eliminate Mtb through innate and adaptive immunity. Therefore, we determined the direct therapeutic efficacy of Rv3463 in a Mtb-infected mouse model. Rv3463-DDA/MPL was injected three times subcutaneously starting from 10 days after intratracheal Mtb infection (Fig. 8A). ESAT-6, which has promising potential as a post-exposure vaccination^28^, was used as a control antigen. The mycobacterial burdens in the lungs and spleens were determined at 2 weeks after the final immunization. Rv3463- and ESAT-6-immunized groups had significantly reduced bacterial loads in the lungs (Rv3463, −0.35log_10_; ESAT-6, −0.14log_10_) and spleens (Rv3463, −0.36log_10_; ESAT-6, −0.16log_10_) (Fig. 8B) when compared to the DDA/MPL control. Rv3463 showed a significantly higher therapeutic efficacy than ESAT-6. Finally, to estimate the role of Rv3463 during infection, the mice were infected with the Rv3463-expressing *M. smegmatis* strain and vector control strain, and then their bacterial burden was determined. Growth of *M. smegmatis* expressing Rv3463 in the lung and spleen (Fig. 8C) was significantly lower than that of the vector control strains. Overall, these results indicate that Rv3463 leads to rapid clearance of bacteria expressing Rv3463, suggesting its potential as a therapeutic vaccine.

## Discussion

The innate immune response plays an important role in protection from TB as it provides the first line of defense against invading pathogens, and can clear the infection in many cases if activated correctly^29^. Macrophages are large mononuclear cells of the innate immune system that function as specialized phagocytic cells which can be activated by inflammatory or various other stimuli^30^. Previously, several mycobacterial proteins that induce the activation of macrophages have been reported^31,32^. However, there have been no reports on the macrophage-activating Mtb proteins with bactericidal activity and their detailed mechanisms. In this study, we found that Rv3463 strongly activates macrophages to enhance phagosomal maturation via PI3K and Ca^2+^ signaling and therefore exhibits therapeutic potential *in vivo*.

Although optimal immune responses are induced during Mtb infection, all hosts do not completely eliminate the bacteria^1^. Therefore, we have been trying to identify macrophage- or dendritic cell-activating proteins with mycobactericidal activity as vaccine targets^15,33^. In this study, the novel macrophage-activating protein, Rv3463, was identified from a multidimensional fraction of Mtb culture filtrate. It is a conserved hypothetical protein with unknown function, and there have been no previous reports on its biological or immunological activities or its interaction partners. The sequence corresponding to the Rv3463 gene is also present in other mycobacterial strains, but represented a non-essential gene. Therefore, further studies are needed to investigate the potential role of Rv3463 in TB pathogenesis. Because for many conserved hypothetical proteins, only the general biochemical function has been predicted, it is necessary to study their exact biological or immunological function through direct experimentation; that being so, hypothetical protein studies can expect various host responses to Mtb^34^. The recombinant Rv3463 protein activated macrophages to induce secretion of pro-inflammatory cytokines including TNF, IL-6, and IL-12, and to upregulate the expression of the surface co-stimulatory molecules as well as MHC molecules. Although production of IL-10, an anti-inflammatory cytokine, was increased in BMDMs stimulated with Rv3463 at a concentration of 5 μg/ml, this could be considered an immune balance action to control the excessive pro-inflammatory cytokine response^35^. It has been reported that several mycobacterial proteins are able to activate immune cells via the TLR2 or TLR4 signaling pathways^36,37^, thereby activating innate immune and inflammatory responses. Our data suggest that Rv3463 interacts with both TLR4 and TLR2, but mainly TLR4, which results in induction of the macrophage activation. In previous studies, we have identified mycobacterial proteins that induce the activation of macrophages or dendritic cells via interaction with TLR4 such as Rv2882c^15^, Rv2299c^36^, Rv0652^38^, and RpfE^39^. It has also been reported that the 38 -kDa protein pstS1 and HSP70 activate pro-inflammatory signals through both TLR2 and TLR4 pathways in macrophages^40,41^. MAPKs, PI3K, and NF-κB are responsible for cellular growth as well as inflammatory and immune responses^32,42,43^. As expected, Rv3463 strongly induced the phosphorylation of ERK1/2, p38, JNK, PI3K, Akt, and NF-κB in macrophages. It is well known that these intracellular signaling molecules activated by the engagement of TLRs are involved in regulation of innate immune responses to mycobacteria^44^.

Virulent mycobacteria can manipulate and interfere with both innate and adaptive immune responses to ensure their survival and persistence^45,46^. Our data showed that a significant production of TNF-α and IL-12 was not induced in Mtb-infected macrophages. Furthermore, it has been reported that IL-12 production was significantly suppressed in macrophages that were pre-exposed to Mtb before IFN-γ stimulation^47^. Phosphorylation of MAPKs, pro-inflammatory cytokine production, and the expression of MHC class or co-stimulatory molecules were significantly increased by Rv3463 stimulation in Mtb-infected macrophages, suggesting that Rv3463 treatment may overcome the Mtb-mediated modulation of innate immune responses. While we have not conducted antigen presentation experiments, it may be expected that Rv3463 improves the antigen presentation ability of macrophages to T cell and T cell activation based on evidence of increased expression of co-stimulatory molecules and MHC by Rv3463 in Mtb-infected macrophages.

A key part of the macrophage defense mechanism against mycobacteria is to kill the phagocytic bacteria through relevant activating processes. Interestingly, Rv3463-activated macrophages, but not those activated by LPS, Ag85 or ESAT-6, significantly suppress Mtb growth. Furthermore, here we confirmed that Rv3463 binds to both TLR2 and TLR4, as demonstrated by the Rv3463-mediated inhibition of Mtb growth, which was abrogated in BMDMs from TLR2^−/−^ or TLR4^−/−^ mice. The ability of Mtb to arrest phagosomal maturation contributes to its survival and persistence within host macrophages^48^. Phagosome biogenesis relies on interactions of the pathogen-containing phagosome with compartments of the endocytic pathway. During phagocytosis, the phagosome recruits the early endosomal marker, small GTPase Rab5, which then recruits type III PI3K VPS34^7^. Production of the lipid regulator PI3P from phosphatidylinositol (PI) is catalyzed by VPS34, which can be reversibly inhibited by LY294002^49^. PI3P is a phagosomal membrane tag that is critical for subsequent phagosomal biogenesis, and inhibition of PI3P production arrests phagosomal maturation^50^. Subsequently, the phagosome acquires the late endosomal marker Rab7 and then fuses with lysosomes containing microbicidal factors such as LAMP1. Confocal analysis for the colocalization of Rab5, VPS34, Rab7, or LAMP1 to Mtb phagosomes suggests that Rv3463 enhances phagosome maturation and fusion with lysosomes in macrophages, which were suppressed by PI3K inhibitors, thereby abrogating Rv3463-mediated bactericidal activity. In fact, several mycobacterial components that block phagosome maturation have been reported; these include Ndk (nucleoside diphosphate kinase), which inactivates Rab5 and Rab7^51^, SapM, which hydrolyzes PI3P into PI^50^, and PtpA, which inhibits phagosome acidification via binding subunit H of V-ATPase^12^. However, there has been no report on a mycobacterial protein that enhances phagosome maturation.

It has been demonstrated that Ca^2+^ affects phagosome biogenesis^50,52^. Ca^2+^ and CaM kinase II are responsible for the accumulation of Rab5 and VPS34 at the phagosomal membrane^53^. Mtb LAM blocks phagosomal maturation by inhibiting the rise in cytosolic Ca^2+^ ^13,54^ and subsequently reduces PI3P production. In this study, Rv3463 caused an increase in cytosolic Ca^2+^ in Mtb-infected macrophages. These responses were not affected by the pre- or post- treatment of Mtb LAM, suggesting that Rv3463-mediated rise in Ca^2+^ did not interfere by inhibition of calmodulin-dependent PI3P production mediated by LAM. Therefore, the detailed mechanism of Ca^2+^ rise induced by Rv3463 should be further investigated.

To evaluate the role of Rv3463 during Mtb infection, we constructed *M. smegmatis* expressing Rv3463, which was more rapidly cleared in macrophages or mice when compared to the vector control strain. However, because Mtb can persist and replicate in host macrophages, it seems likely that the role of Rv3463 activity in the enhancement of phagolysosomal fusion in TB pathogenesis might be minor and overshadowed by other virulence factors. In fact, Mtb contains virulence components that are responsible for mycobacterial survival in macrophages, such as LAM or PtpA. In addition, it contains some components that can induce the host protective response through stimulation of the immune system, such as immunodominant T cell-stimulating proteins, which have been used for development of the TB vaccine^15,33^. Based on our results, we expected that Rv3463-mediated macrophage activation may contribute to protection against Mtb infection. Therefore, we tested the relation between bactericidal activity of Rv3463 and therapeutic efficacy *in vivo*. We found that Rv3463 exhibited a significant direct therapeutic efficacy when compared to ESAT-6 in a short-term infection model using avirulent Mtb H37Ra, indicating that the capability for eliminating intracellular Mtb correlated with the therapeutic effect *in vivo*. It has also been demonstrated that only ESAT-6 or ESAT-6-containing fusion vaccines show significant protection in a latent TB mouse model, but ESAT-6 has no therapeutic effect in chronically infected mice^28^. In our model, ESAT-6 showed a significant therapeutic effect when compared to adjuvant controls. We previously demonstrated that Mtb H37Ra can be used to test vaccine efficacy in a mouse model^15^. However, further studies are needed to investigate the detailed therapeutic efficacy and mechanism of Rv3463 in acute or chronic infection model using virulent Mtb H37Rv.

Taken together, our study suggests that Rv3463 induces a bactericidal effect in Mtb-infected macrophages through phagosome maturation enhanced by the PI3K and Ca^2+^ signaling pathways, and exhibits therapeutic effects *in vivo*. Our findings reveal a novel target for TB therapy, which induces host and cellular immunity, which would provide a rational design for post-exposure TB vaccines or secondary immunotherapy that can be used with chemotherapy.

## Methods

### Ethics statement

All animal experiments were performed in accordance with the Korean Food and Drug Administration (KFDA) guidelines for animal care and use. Animals work was done in accordance with procedures that were approved by the Institutional Animal Care and Use Committee of Chungnam National University, South Korea (Permit number: CNU-00284).

### Mice

Specific pathogen-free, 5–6 week-old, female C57BL/6 (H-2Kb and I-Ab), C57BL/6J TLR2 knockout (TLR2^−/−^; B6.129-Tlr2tm1Kir/J), and C57BL/10 TLR4 knockout (TLR4^−/−^; C57BL/10ScNJ) mice were purchased from the Jackson Laboratory (Bar Harbor, ME, U.S.A) and used in all experiments. TLR2/4 double knockout (TLR2/4^−/−^) mice were obtained from Chonnam National University (Gwangju, Korea). The mice were maintained under barrier conditions in a biohazard animal room at the Medical Research Center of Chungnam National University (Daejeon, Korea). The animals were fed a sterile commercial mouse diet with *ad libitum* access to water under standardized light-controlled conditions (12-h light and 12-h dark periods). The mice were monitored daily, and none of the mice showed any clinical symptoms or illness during this experiment.

### Cell culture

Bone marrow-derived macrophages (BMDMs) were generated by flushing bone marrow cells from femurs and tibias, and cultured for 6 days in Dulbecco’s modified Eagle’s medium (Welgene Co., Daegu, Korea) containing 10% fetal bovine serum (FBS) (Welgene), 50 ng/ml mouse macrophage colony stimulating factor (M-CSF) (R&D Systems, Minneapolis, MN, U.S.A) and 1% antibiotics (Welgene), and incubated at 37 °C in a 5% CO_2_ atmosphere.

THP-1 cells (KCLB 40202) were purchased from Korean Cell Line Bank (Seoul, Korea). THP-1 cells were cultured in RPMI 1640 supplemented with 10% FBS, 1 mM sodium pyruvate (Sigma, St. Louis, MO, U.S.A), 1% nonessential amino acids (Lonza, Basel, Switzerland), 100 unit/ml penicillin/streptomycin (Welgene), and incubated at 37 °C in a 5% CO_2_ atmosphere_._

### Bacterial strains

Mtb H37Rv (ATCC 27294, Mtb), Mtb H37Ra (ATCC 25177) and *M. smegmatis* strain mc^2^155 were purchased from American Type Culture Collection (ATCC, Manassas, VA). Mycobacteria were grown in 7H9 medium supplemented with 0.5% glycerol, 0.05% Tween-80 (Sigma), 10% oleic acid, albumin, dextrose, and catalase (OADC; BD Biosciences, San Jose, CA, U.S.A).

The H37Rv-expressing red fluorescent protein (Mtb-RFP) was constructed in this study. In briefly, Mtb-RFP fragment was amplified from pRSET-RFP1 with primers (forward, -5′-ATGGATCCTATGGCCTCCTCCGAG-3′ and reverse, -5′-TTGAATTCTTAGGCGCCGCTCGAG-3′), and inserted to *BamH*I and *EcoR*I sites of pMV261 vector. The constructed plasmid pMV261-RFP was electroporated into Mtb H37Rv and selected for kanamycin resistance and for the expression of red fluorescence. The Mtb-RFP strain was cultivated in 7H9 medium containing 50 μg/ml kanamycin (Sigma). Experiments involving Mtb were carried out in a Biosafety Level 2 (BSL2) laboratory.

### Expression and production of recombinant protein

To produce recombinant Rv3463 protein, the corresponding gene was amplified by PCR using Mtb H37Rv ATCC 27294 genomic DNA as the template and the following primers: *Rv3463* forward, 5′-CATATGACCAATTGTGCCGCCGGCAAA-3′, and reverse, 5′-AAGCTTAGTCAGTCGGAGCGGCTTCGC-3′; *ESAT6* forward, 5′-AAGCTTATGACAGAGCAGCAGTGGAAT-3′, and reverse, 5′-CTCGAGTGCGAACATCCCAGTGACGTT-3′; *Ag85* forward, 5′-GAATTCGATGACAGACGTGAGCCGAAAG-3′, and reverse, 5′-AAGCTTGCCGGCGCCTAACGAACTCTG-3. The PCR product of *Rv3463* was cut with *Nde*I and *Hind*III, *ESAT6* was cut with *Hind*III and *Xho*I, *Ag85* was cut with *EcoR*I and *Hind*III. The products were inserted into pET22b (+) vector (Novagen, Madison, WI, U.S.A) and the resultants were sequenced. The recombinant plasmids containing *Rv3463*, *ESAT6*, and *Ag85* were transformed into *E. coli* BL21 cells by heat-shock for 1 min at 42 °C. The recombinant proteins were prepared as previously described^15^.

### Antibodies and reagents

An Endotoxin filter (END-X) and endotoxin removal resin (END-X B15) were acquired from the Associates of Cape Cod (East Falmouth, MA, U.S.A). Fluorescein isothiocyanate (FITC)-annexin V/PI kits (556547) were purchased from BD Biosciences. LPS from *Escherichia coli* O111:B4 (tlrl-eblps) and palmitoyl-3-Cys-Ser-(Lys)4 (Pam3CSK4, tlrl-pms) was purchased from InvivoGen (San Diego, CA, U.S.A). Mouse TNF-α (88-7324-77), IL-6 (88-7064-77), IL-10 (88-7105-77) and IL-12p70 (88-7121-77), ELISA kits were obtained from eBioscience (San Diego, CA, U.S.A). Phycoerythrin (PE)-conjugated mAbs directed against CD80 (16-10A1), CD86 (GL1), MHC class I (34-1-2S) and MHC class II (I-A/I-E, M5/114.15.2), and allophycocyanin-conjugated mAb directed against F4/80 (BM8) were purchased from eBioscience. Anti-TLR2 (H-175, sc-10739), anti-TLR4 (25, sc-293072) and anti-histidine (His, sc-8036) Ab were purchased from Santa Cruz Biotechnology (Paso Robles, CA, U.S.A). Horseradish peroxidase-conjugated anti-mouse IgG (401215) and anti-rabbit IgG (401353) were purchased from Calbiochem (San Diego, CA, U.S.A). Anti-phosphorylated ERK1/2 Ab (T202/Y204, 9101), anti-phosphorylated p38 Ab (T180/Y182, 9211), anti-phosphorylated JNK monoclonal Ab (T183/Y185, 4671), anti-phosphorylated PI3K Ab (Y458, 4228), anti-phosphorylated Akt (S473, 9271), anti-phosphorylated IκB-α Ab (S32, 14D4, 2859), anti-IκB-α Ab (44D4, 4812), β-actin Ab (13E5, 4970), and anti-NF-κB p65 Ab (D14E12, 8242) were obtained from Cell Signaling Technology (Danvers, MA, U.S.A). Specific inhibitors of ERK1/2 (U0126, 662005), p38 (SB203580, 559389), JNK (SP600125, 420119), NF-κB (BAY 11-7082, 196870), PI3K (Ly294002, 440202) and wortmannin (681675) were purchased from Calbiochem. Signal silence control siRNA (6568) and signal silence PI3K siRNA I (6912) were purchased from Cell Signaling Technology. Lipofectamine 2000 was obtained from Invitrogen (Carlsbad, CA, U.S.A). For immunofluorescence analysis anti-Rab5 Ab (C8B1, 3547), anti-Rab7 (D95F2, 9367) and anti-VPS34 (D9A5, 4263) were purchased from Cell Signaling Technology. Anti-LAMP1 Ab (1D4B, sc-19992) was obtained from Santa Cruz Biotechnology. DAPI (D3571) was obtained from Molecular Probes (Eugene, OR, U.S.A). Alexa-488 goat anti-mouse IgG (A11001), Alexa-488 goat anti-rabbit IgG (A11008), and Alexa-568 goat anti-mouse IgG (A11031) were purchased from Thermo Fisher Scientific (Waltham, MA, U.S.A) and Alexa 488 goat anti-rat IgG (ab150165), and 350 goat anti-rabbit IgG (A11046) were obtained from Abcam (Abcam, Cambridge, UK). Texas Red®-X phalloidin (T7471) was purchased Molecular Probes. Rapamycin (553210) was purchased from Calbiochem. Anti-LC3A/B (L8918) for immunofluorescence and immunoblotting was purchased from Sigma-Aldrich. For Ca^2+^ measurement Fluo-4/AM (F14201) and Fura-2/AM (F1221) were purchased from Thermo Fisher Scientific, Mtb LAM antigen (DAGA-168) was purchased from Creative Diagnostics (CD, Shirley, NY, U.S.A), and ionomycin (19657) was obtained from Sigma- Aldrich.

### Cytotoxicity analysis

To confirm the toxicity of Rv3463, BMDMs (5 × 10^5^/well) were treated Rv3463 (5 μg/ml) or LPS (100 ng/ml) for 24 h. After incubation, the harvested BMDMs were washed with PBS, stained with FITC-Annexin V and PI for 15 min, and analyzed using FACSCanto flow cytometer (BD Biosciences).

### ELISA for cytokines

The culture supernatants were collected from BMDMs (1 × 10^5^/well) stimulated with various concentrations of Rv3463, LPS, Ag85 or ESAT-6. Sandwich ELISAs for detecting the cytokines in the culture supernatants were performed as recommended by the manufacturer (eBioscience). Plates were read on a Vmax kinetic microplate reader (Molecular Devices Co., Sunnyvale, CA, U.S.A) at 450 mm.

### Analysis of expression of cell-surface molecules

BMDMs (1 × 10^5^/well) were treated with each stimulant for 24 h, harvested, washed with PBS, and re-suspended in. The cells were pre-incubated with 0.5% BSA in PBS for 30 min and washed with PBS. Cell surface molecule staining was performed using specifically labeled fluorescent-conjugated Abs, and staining intensity was determined using flow cytometry (FACSCanto) and data were analyzed using FlowJo data analysis software (BD Bioscience).

### Immunoprecipitation

THP-1 cells (1 × 10^7^/well) were lysed with lysis buffer [50 mM Tris HCl, pH 8.0; 137 mM NaCl; 1 mM EDTA; 1% (vol/vol) Triton X-100; 10% (vol/vol) glycerol; 1 mM PMSF; 1 μg/mL each of aprotinin, leupeptin, and pepstatin; 1 mM Na_3_VO_4_; 1 mM NaF; and proteinase inhibitor cocktail tablet (Roche, Basel, Switzerland)] for 20 min on ice. The lysate was cleared of cell debris by centrifugation at 17,000 xg for 10 min at 4 °C. The cell lysate and 20 μg His-tagged Rv3463 protein were mixed and incubated at 4 °C for 6 h, and then His-tagged Rv3463 (His)-, TLR2- and TLR4-associated proteins were immunoprecipitated by incubation with Ni-NTA Agarose (Qiagen, Hilden, Germany) or Dynabeads®Protein A (Thermo Fisher Scientific) for 24 h at 4 °C after incubation with an anti-mouse IgG Ab as a control Ab for anti-Rv3463 (His), anti-rabbit IgG Ab as a control Ab for the anti-TLR2 and anti-TLR4 for 4 h at 4 °C. The beads were collected, washed and boiled in 5x sample buffer for 5 min. The bound proteins were analyzed on by immunoblotting with anti-TLR2, anti-TLR4, and anti-His Abs.

### Immunoblotting analysis

After stimulation with 5 μg/ml Rv3463, cultured cells were lysed with lysis buffer and whole-cell lysate samples were resolved on SDS-polyacrylamide gels. Subsequently, the proteins were transferred onto a nitrocellulose membrane. The membranes were blocked in 5% skim milk and incubated with the Abs for 24 h at 4 °C, followed by incubation with HRP-conjugated secondary Abs for 1 h at room temperature. Epitopes on target proteins recognized specifically by Abs were visualized by using the ECL advance kit (GE Healthcare, Little Chalfont, UK).

### Anti-Rv3463 antibody

To obtain antiserum against Rv3463, BALB/c mice were immunized intraperitoneally with 25 μg purified recombinant Rv3463 emulsified in incomplete Freund’s adjuvant. Mice were injected with antigen three times at 2-week intervals, and the serum was collected 1 week after the final immunization.

### Transformation of recombinant Rv3463-expressing *M. smegmatis* strains

Transformation of recombinant Rv3463-expressing *M. smegmatis* strains was performed as described previously^55^. In brief, *M. smegmatis* strain mc^2^155 was grown in 7H9 medium supplemented with 10% OADC and 0.05% Tween-80. Cells were then harvested and washed twice with cold 10% glycerol and resuspended in the same buffer. Competent cells were electroporated using the Gene Pulser apparatus (Bio-Rad, Hercules, CA, U.S.A) with standard settings. Transformants were selected on 7H10 agar plates containing 50 μg/ml kanamycin. Plates were incubated at 37 °C for 3–4 days to obtain the recombinant strains.

### Analysis of bacterial growth

BMDMs were infected with Mtb-RFP (1 × 10^5^/well) for 4 h and then treated with each stimulant. Subsequently, the cells were harvested, washed with PBS, and re-suspended in medium. The cells were pre-incubated with 0.5% BSA in PBS for 30 min and washed with PBS. Staining of cell surface molecules was performed using F4/80 Abs, and staining intensity was determined using flow cytometry (FACSCanto) and data were analyzed using FlowJo data analysis software (BD Bioscience).

For *in vitro* infections with Mtb, BMDMs (1 × 10^5^/well) were infected with Mtb at a multiplicity of infection (MOI) of 1 for 4 h at 37 °C, 5% CO_2_. Amikacin (200 μg/ml; Sigma) was added to each well and cell were incubated for 2 h to kill extracellular bacteria, and then the cells were washed three times with PBS and treated with Rv3463 or other proteins for the indicated additional time period. After incubation, the cells were lysed with sterile distilled water for 30 min. The lysates were serially diluted and plated onto 7H10 agar plates to determine the “input” bacterial numbers.

For *in vitro* infections with *M. smegmatis*, BMDMs (1 × 10^5^/well) were infected with *M. smegmatis* at a MOI of 10 for 4 h at 37 °C, 5% CO_2_. Gentamicin (100 μg/ml; Sigma) was added for 2 h to remove any remaining extracellular bacteria, and then in medium with 10% FBS containing 10 μg/ml gentamicin for the indicated additional time period. After incubation, bacterial load measurement was performed as described above.

### AlamarBlue assay

AlamarBlue assay was performed to measure the viability of *M. smegmatis*. The bacterial culture were adjusted to an OD600 of 0.05 and cultured with 7H9 medium containing 10% OADC and 0.05% Tween-80 for the indicated additional time period.at 37 °C. The absorbance signal (570 nm and 600 nm) were measured by Vmax kinetic microplate reader.

### Colocalization of phagosomes and phagolysosomes

To observe the colocalization of Mtb-containing phagosomes, we performed confocal microscopy as described previously^55^. BMDMs (2 × 10^5^/well) were prepared in 12-well culture dishes that contained 18 mm diameter round glass coverslips. The cells were then infected with Mtb-RFP at a MOI of 1 for 4 h at 37 °C in a 5% CO_2_ incubator and incubated with Rv3463 or ESAT-6. After 72 h incubation, the cells were stained with anti-Rab5, anti-VPS34, anti-Rab7 or anti-LAMP1 antibodies imaged under the confocal microscope.

### *M. smegmatis* staining

*M. smegmatis* staining was carried out as described previously. In brief, the washed bacteria (1 × 10^8^ cells/ml) were suspended in 500 µl of PBS, mixed with 10 µM carboxyfluorescein diacetate succinimidyl ester (CFSE; C34554, Invitrogen) incubated for 10 min at room temperature, and washed twice in PBS supplemented with 5% FBS. Before infection, bacteria were resuspended using bath sonication and vortexing.

### Autophagy analysis

LC3 punctate staining was quantified in triplicate from at least 50 randomly chosen cells. LC3-II protein levels were evaluated by immunoblotting using an antibody against LC3A/B (Sigma).

### Cell transfections

BMDMs (5 × 10^5^/well) were transfected with a control siRNA (siCON, 50 nM) and PI3K siRNA (siPI3K, 50 nM) using Lipofectamine 2000 for 24 h according to the manufacture’s instructions. The transfection medium was then replaced with normal medium.

### Intracellular Ca^2+^ measurements

Intracellular Ca^2+^ was measured as described previously^13,56^. BMDMs (5 × 10^5^/well) grown on coverslips were loaded with the Ca^2+^ indicator Fluo-4/AM (10 μM, 30 min; for confocal measurements) or Fura-2/AM (10 μM, 30 min; for kinetic measurements) before treatment of LAM (20 μg/ml) in Hank’s balanced salt solution (HBSS), according to the manufacturer’s protocol (Molecular Probe). The cells were washed twice with HBSS, infected with Mtb (MOI = 1) for 2 h, washed twice in PBS, and incubated with Rv3463 (5 μg/ml), LAM (20 μg/ml) or mixture of Rv3463 and LAM in for the indicated time period. Confocal images were obtained using a Leica TCS SP8 confocal laser scanning microscope (Leica), with an excitation wavelength of 488 nm and emission at 500–550 nm. For kinetic measurements, cells were collected before ionomycin (500 nM) or Rv3463 (5 μg/ml) treatment as well as every 5 s for 3 min after treatment as using a Nikon Eclipse Microscope. Fura-2/AM was excited at 380 nm (calcium-free form) and 340 nm (calcium-bound form), and emission was detected at 510 nm.

### Mycobacterial infection *in vivo*

For testing the therapeutic efficacy of Rv3463, C57BL/6 mice were injected via the intratracheal (I.T) instillation with Mtb H37Ra (1 × 10^6^ CFU/mouse). After 10 days post-infection, mice were immunized with Rv3463 or ESAT-6 (25 μg) three times at 10 days intervals with dimethyldioctadecylammonium (DDA) liposomes (50 μg/injection) containing monophosphoryl lipid-A (MPL, 5 μg/injection) (DDA/MPL). Two weeks after the final immunization, mice were sacrificed.

*M. smegmatis* infection *in vivo* was performed as described previously^57^. C57BL/6 mice were intravenously (tail vein) injected with different strains of *M. smegmatis* (using 1 × 10^6^ CFU/mouse) for 1, 3 or 7 days. At each time point (1, 3 or 7 days post infection), mice were sacrificed.

For measurement of the bacterial burden in the lung and spleen, the organs were homogenized in 1 ml PBS and serial dilutions of the homogenates were plated on 7H10 agar plates for *M. tuberculosis* H37Ra or on 7H10 agar plates containing 50 μg/ml kanamycin for *M. smegmatis*. CFUs were determined after incubation at 37 °C.

### Statistical analysis

All experiments were repeated at least 3 times with consistent results. The levels of significance for comparisons between samples were determined by Tukey’s multiple comparison test distribution or two-way ANOVA using statistical software (GraphPad Prism Software, version 4.03; GraphPad Software, San Diego, CA). The data in the graphs are expressed as the mean values ± SD; *p < 0.05, **p < 0.01 or ***p < 0.001 were considered statistically significant.

## Supplementary information

Mycobacterium tuberculosis Rv3463 induces mycobactericidal activity in macrophages by enhancing phagolysosomal fusion and exhibits therapeutic potential
