# Supplementary material for: Mycobacterium tuberculosis Rv3463 induces mycobactericidal activity in macrophages by enhancing phagolysosomal fusion and exhibits therapeutic potential
Source: Sci Rep. 2019 Mar 12;9:4246. doi: 10.1038/s41598-019-38982-0 (PMC6414722; doi:10.1038/s41598-019-38982-0)
Supplement: Supplementary file 1 — Mycobacterium tuberculosis Rv3463 induces mycobactericidal activity in macrophages by enhancing phagolysosomal fusion and exhibits therapeutic potential [file 41598_2019_38982_MOESM1_ESM.docx]

**Supplementary information**

***Mycobacterium tuberculosis* Rv3463 induces mycobactericidal activity in macrophages by enhancing phagolysosomal fusion and exhibits therapeutic potential**

Hye-Soo Park ^1^, Yong Woo Back ^1^, Ki-Won Shin ^1^, Hyun Shik Bae ^1^, Kang-In Lee ^1^, Han-Gyu Choi ^1^, Seunga Choi ^1^, Hwang-Ho Lee ^2^, Chul Hee Choi ^1^, Jeong-Kyu Park ^1^, and Hwa-Jung Kim^*, 1^

^1^Department of Microbiology and Department of Medical Science, College of Medicine, Chungnam National University, Daejeon, Republic of Korea

^2^Department of Microbiology and Immunology, Chonbuk National University Medical School, Jeonju, Republic of Korea

^*^Address correspondence to Hwa-Jung Kim, M.D., Ph.D., Department of Microbiology, College of Medicine, Chungnam National University, 266 Munwha-Dong, Jung-Ku, Daejeon 301-747, South Korea; Tel.: +82-42-580-8242; Fax: +82-42-585-3686, E-mail: [hjukim@cnu.ac.kr](mailto:hjukim@cnu.ac.kr)

**Supplementary Figure**

**
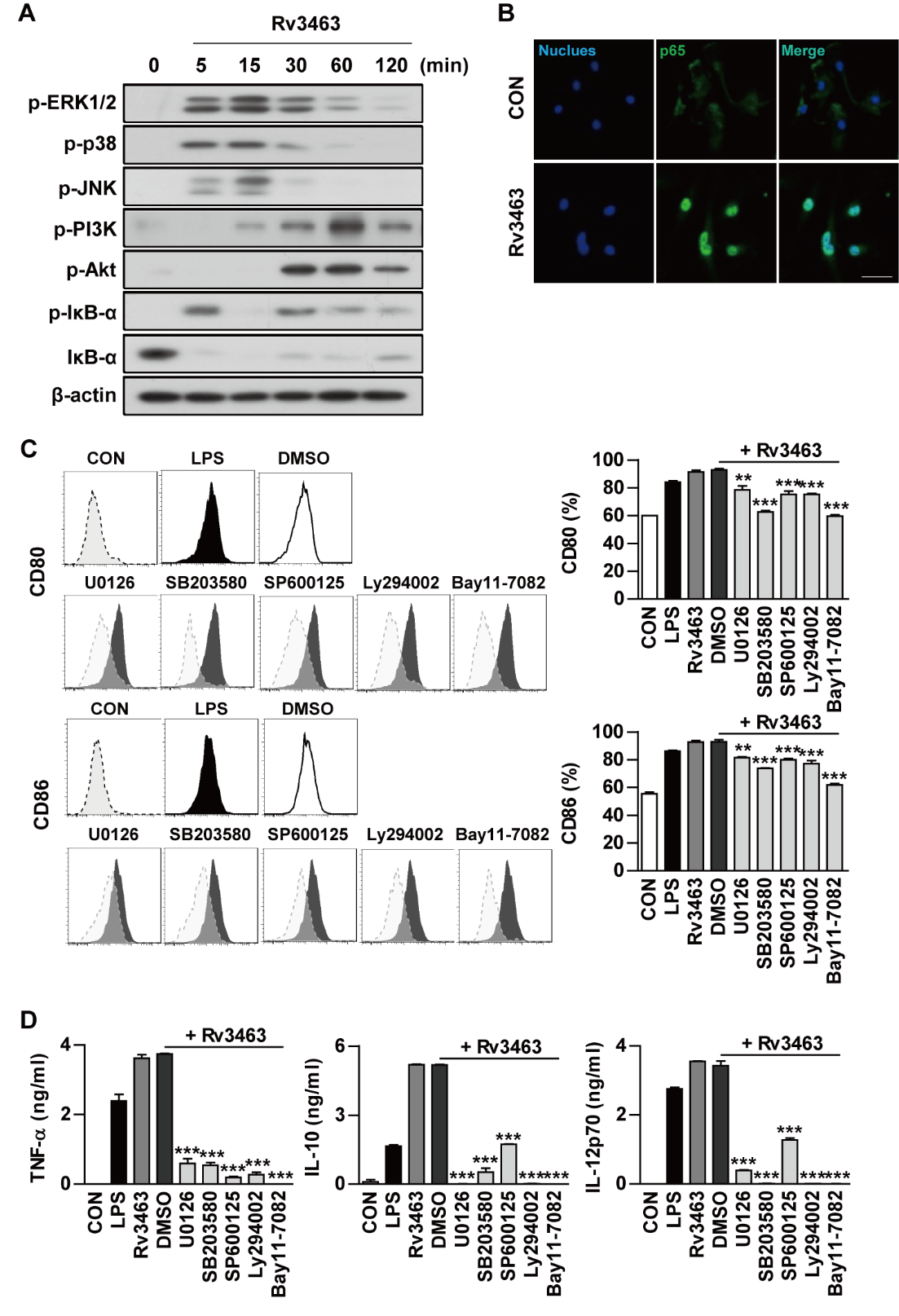
**

**Supplementary Figure 1. MAPK, PI3K and NF-κB pathways are involved in Rv3463-mediated macrophage activation.**

(A) BMDMs stimulated with Rv3463 for the times indicated were lysed, and the proteins in the total cell lysate were separated by SDS-PAGE, followed by immunoblot analysis using antibodies against phospho-ERK1/2, phospho-p38, phospho-JNK, phospho-PI3K, phospho-Akt, phospho-IκB-α, IκB-α, and β-actin. This image is representative of three experiments showing similar results. (B) BMDMs were plated in covered glass chamber slides and treated with Rv3463 for 1 h, and the immunoreactivity of the p65 subunit of NF-κB in cells was determined by immunofluorescence. Scale bar, 10 μm. BMDMs were pretreated with pharmacological inhibitors of ERK (U0126, 10 μM), p38 (SB203580, 20 μM), JNK (SP600125, 10 μM), PI3K (LY294002, 20 μM), NF-κB (BAY11-7082, 5 μM), or DMSO (vehicle control) for 1 h prior to the treatment of Rv3463 (5 μg/ml). After 24 h, the amounts of TNF-α, IL-10, and IL-12p70 in the culture medium were measured by ELISA (C). The mean ± SD are shown for three independent experiments. The expressions of CD80 and CD86 were analyzed by flow cytometry (D). Bar graphs show the percentages (mean ± SD of three separate experiments) for each surface molecule on the F4/80^+^ cells. ***p* < 0.01 or ****p* < 0.001 for each inhibitor treatment compared to Rv3463-treated controls.


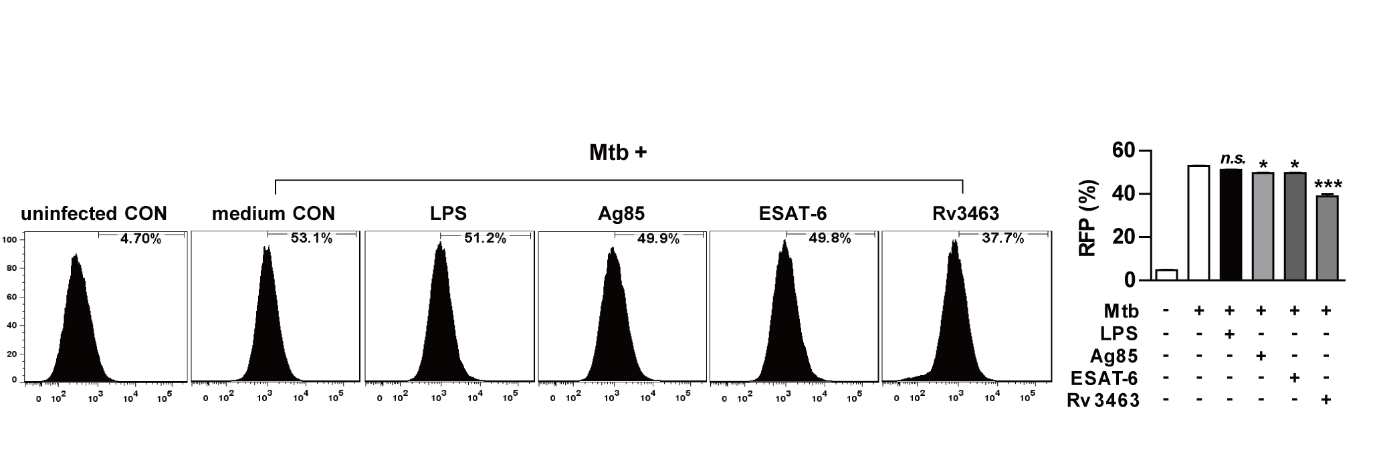
**Supplementary Figure 2. Effect of Rv3463 on *Mycobacterium tuberculosis* (Mtb) growth in macrophages.**

Bone marrow-derived macrophages (BMDMs) were infected with Mtb-RFP at a multiplicity of infection of 1 for 4 h, then incubated with or without 5 μg/ml Rv3463, 100 ng/ml LPS, 5 μg/ml Ag85 or 2 μg/ml ESAT-6 for 72 h. Fluorescence intensity of the cells infected with Mtb-RFP was analyzed by flow cytometry. Graphs shown in each panel are representative of three experiments. Bar graphs show the mean values ± SD (*n* = 3). **p* < 0.05 or ****p* < 0.001 for treatments compared to Mtb infection only controls. *n.s*., no significant difference.


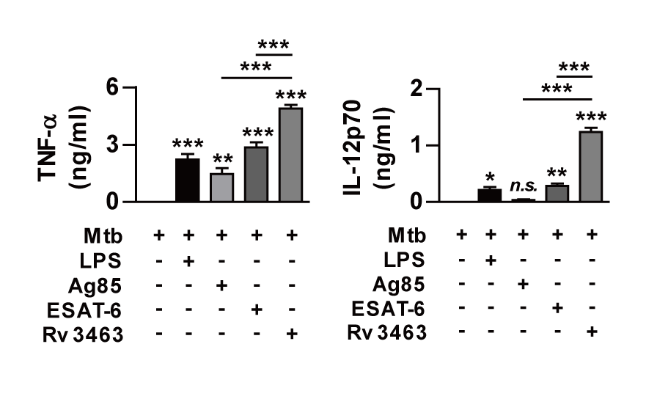


**Supplementary Figure 3. Rv3463-mediated cytokine production in Mtb-infected macrophages.**

Bone marrow-derived macrophages (BMDMs) were infected with Mtb at a multiplicity of infection (MOI) of 1 for 4 h, and then further treated with amikacin to kill extracellular bacteria for 2 h, washed three times, and incubated with or without 5 μg/ml Rv3463, 100 ng/ml LPS, 5 μg/ml Ag85, or 2 μg/ml ESAT-6 for 72 h. The production of TNF-α and IL-12p70 in culture supernatants at 72 h was measured by ELISA. The data shown are mean values ± SD (*n*=3). **p* < 0.05, ***p* < 0.01, and ****p* < 0.001 for each-treatment compared. *n.s*., no significant difference.


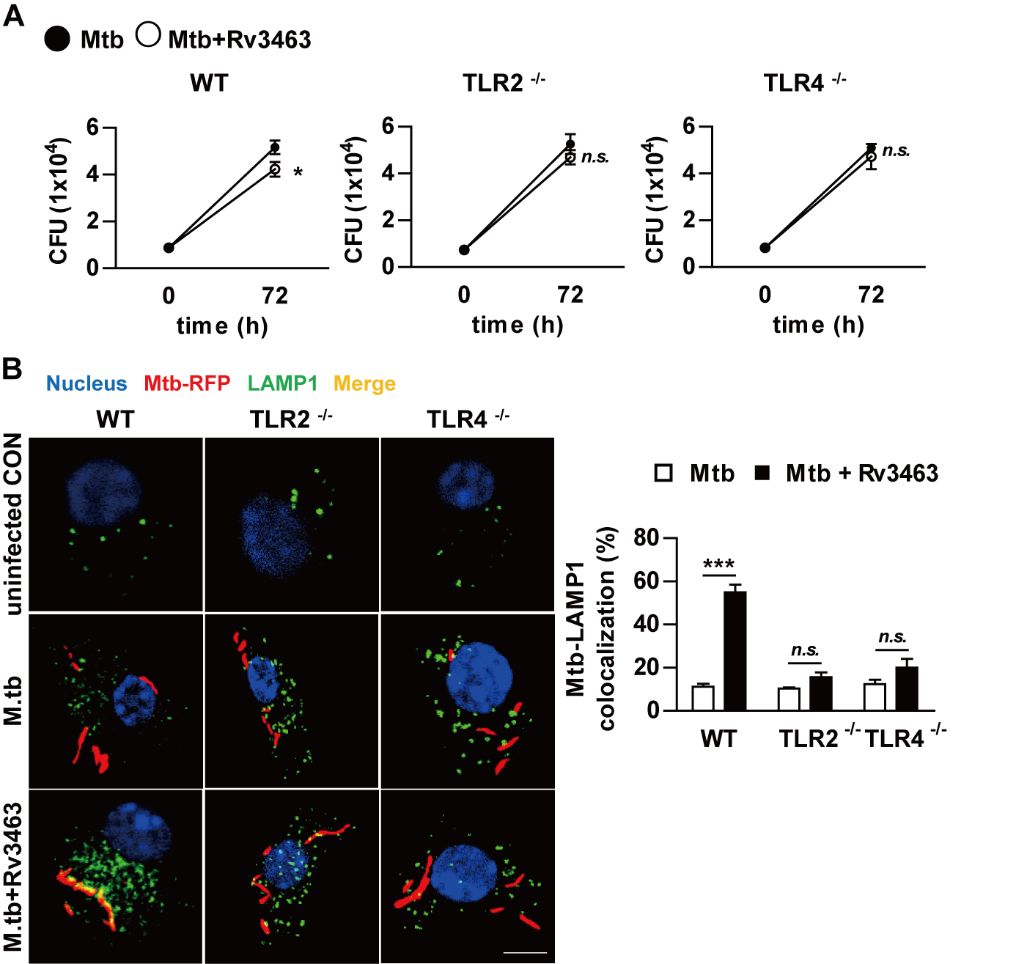


**Supplementary Figure 4. Rv3463-mediated bactericidal activity and phagolysosome fusion was not observed in bone marrow-derived macrophages (BMDMs) from TLR2^-/-^, and TLR4^-/-^ mice .**

A Bone marrow-derived macrophages (BMDMs) derived from WT, TLR2^–/–^, and TLR4^–/–^ mice were infected with Mtb at a multiplicity of infection (MOI) of 1 for 4 h, and then further treated with amikacin to kill extracellular bacteria for 2 h, washed three times, and incubated with or without 5 μg/ml Rv3463 for 72 h. Intracellular Mtb growth was determined by plating the cell lysates on 7H10 agar for 0 to 72 h. All data shown are the mean ± SD (*n* = 3). **p* < 0.05 for treatment compared to infection only controls. (B) BMDMs derived from WT, TLR2^–/–^, and TLR4^–/–^ mice were infected with Mtb-RFP and then incubated with or without 5 μg/ml Rv3463 for 72 h. Mtb phagosome and Lamp1 colocalization was visualized by laser-scanning confocal microscopy as described in the legend of Fig. 5. Scale bar, 10 μm. ****p* < 0.001 for Rv3463 treatment compared. *n.s*., no significant difference.


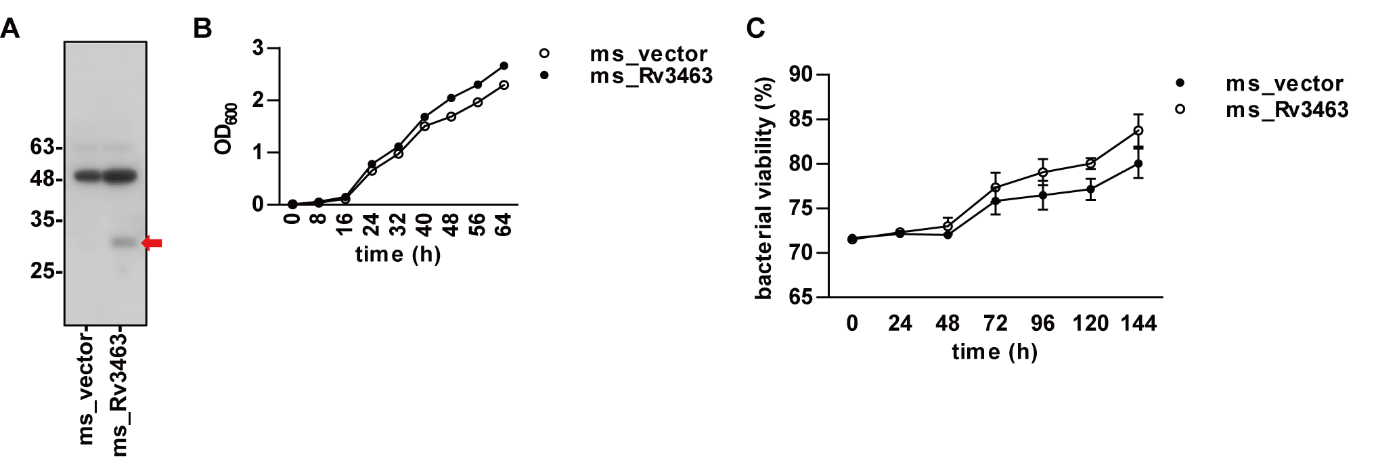


**Supplementary Figure 5. Confirmation of the expression of Rv3463 in recombinant *Mycobacterium smegmatis* and growth rate *in vitro*.**

(A) Total extracts from *M. smegmatis* vector control strain and *M. smegmatis* expressing Rv3463 were subjected to SDS-PAGE and analyzed by western blot using anti-His antibodies. Rv3463 protein band shows as an arrow. (B) Growth of both strains in 7H9 liquid medium was monitored by determining OD_600_ with intervals of 8 h. (C) Rv3463 expressing M. smegmatis (ms_Rv3463) and vector control strain (ms_vector) were cultured for the indicated additional time. After the culture, 0.02% alamarBlue reagent was added to the culture and incubated for 4 h at 37℃. Reduction of alamarBlue reagent was measured by absorbance intensity.

**
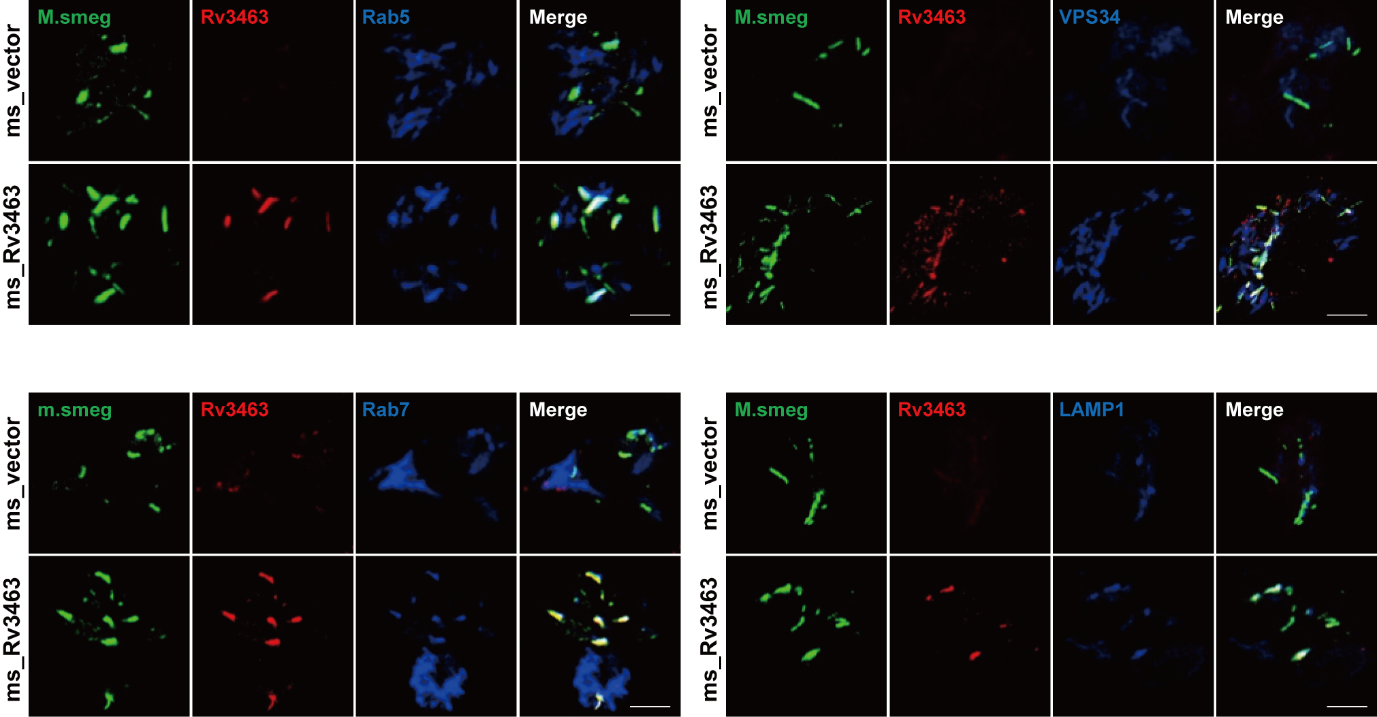
**

**Supplementary Figure 6. Enhancement of the phagosomal maturation was induced in bone marrow-derived macrophages (BMDMs) infected with *M. smegmatis* overexpressing Rv3463.**

BMDMs were infected with CFSE-stained *M. smegmatis* (green) expressing Rv3463 or vector control strain at a multiplicity of infection of 10 for 4 h, treated with gentamycin, and then washed. After 48 h, the cells immunolabeled with anti-Rab5, anti-VPS34, anti-Rab7, or anti-LAMP1 antibodies were further stained with Alexa 350-conjugated goat anti-rabbit IgG (blue). Rv3463 was stained with anti-His antibodies and Alexa 568-conjugated goat anti-mouse IgG (red), and then analyzed by laser-scanning confocal microscopy. Scale bar, 10 μm.


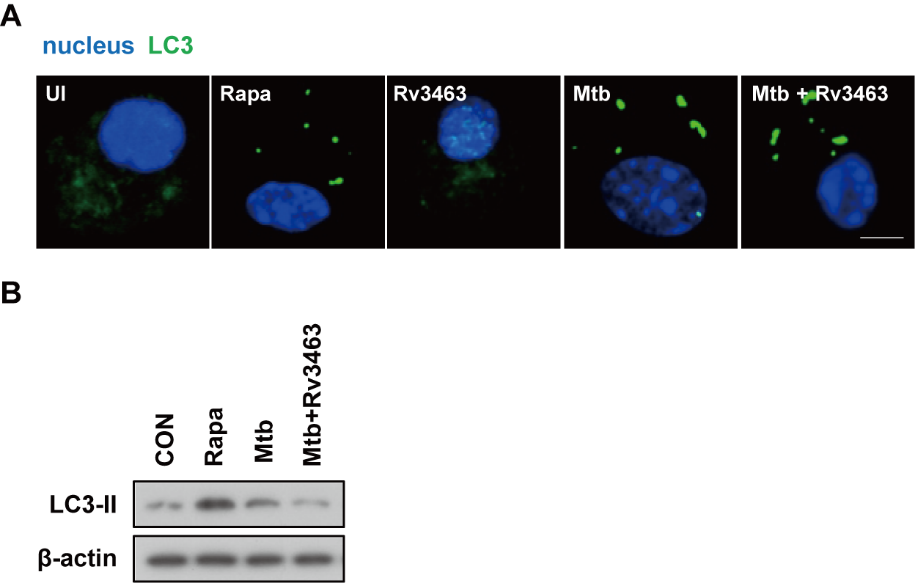


**Supplementary Figure 7.** **Autophagy was not induced by Rv3463.**

(A) Bone marrow-derived macrophages (BMDMs) were incubated with Mycobacterium tuberculosis (Mtb) at a multiplicity of infection of 1 for 4 h, then incubated with or without 5 μg/ml Rv3463 for 4 h, fixed with 4% paraformaldehyde, and immunolabeled with an anti-LC3 and Alexa 488-conjugated goat anti-rabbit IgG (green) antibodies. The cells were stained with DAPI to visualize the nuclei (blue) and then analyzed for LC3 molecules by laser-scanning confocal microscopy. Scale bar, 10 μm. (B) The cell lysates from the BMDMs treated by the indicated conditions were subjected to western blot analysis using antibodies against LC3 and β-actin. Rapamycin (Rapa, 200 nM) treatment for 4 h was used as a positive control.

**
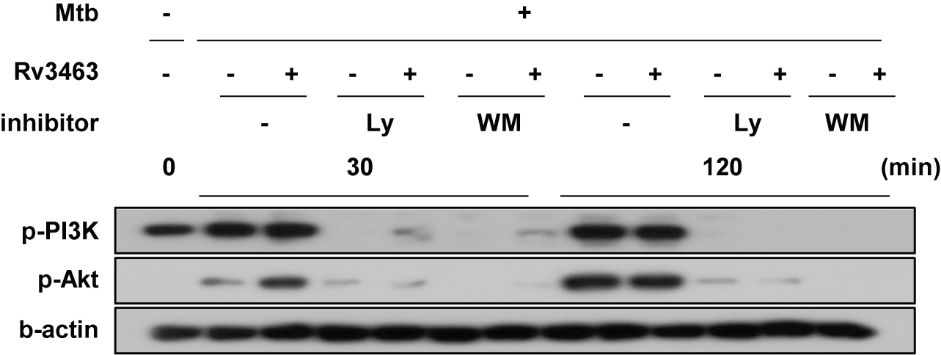
**

**Supplementary Figure 8.** **Confirmation of the activities of PI3K inhibitors.**

Bone marrow-derived macrophages (BMDMs) preincubated with pharmacological inhibitors of PI3K (20 μM LY294002 [Ly] or 200 nM Wortmannin [WM]) for 1 h were infected with *Mycobacterium tuberculosis* Mtb for 2 h, and then further incubated with or without 5 μg/ml Rv3463 for the indicated time period. Protein expressions of phospho-PI3K, phospho-Akt and β-actin in the BMDMs were analyzed by western blot analysis. The image is representative of three experiments showing similar results.


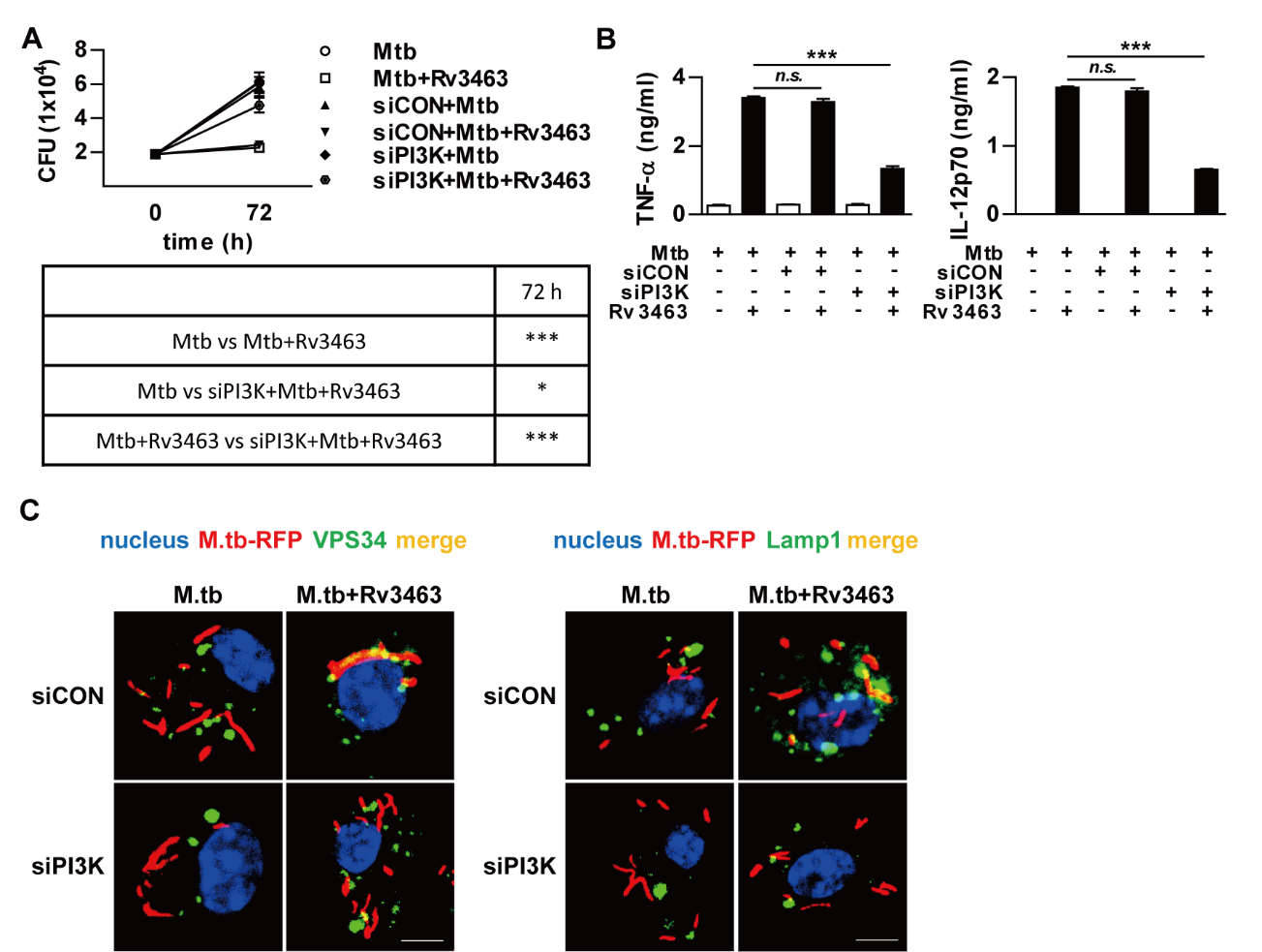


**Supplementary Figure 9. PI3K knockdown interferes the Rv3463-mediated activities.**

Bone marrow-derived macrophages (BMDMs) were transfected with PI3K siRNA (siPI3K) or nonspecific siRNA as a control (siCON) and were infected with Mtb (A, B) or Mtb-RFP (C) at a multiplicity of infection of 1 for 4 h, treated with amikacin, washed, and incubated with or without 5 μg/ml Rv3463 for 72 h. (A) Intracellular Mtb growth in the cells was determined at 0 and 72 h. **p* < 0.05 and ****p* < 0.001 for treatment compared to each controls. (B) TNF-α or IL-12p70 production in culture supernatants were measured by ELISA. *n.s*., no significant difference. (C) Colocalization of VPS34 or Lamp1 molecules (green) with Mtb (red) in the treated BMDMs were analyzed by laser-scanning confocal microscopy. The cells were stained with DAPI to visualize the nuclei (blue).


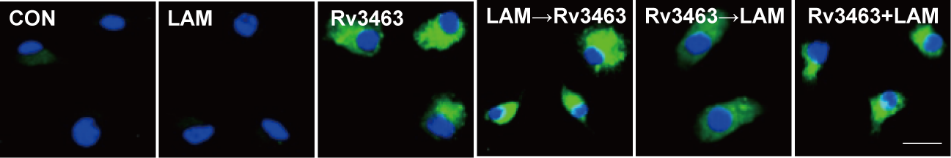


**Supplementary Figure 10. Rv3463 increases the intracellular Ca^2+^ in *Mycobacterium tuberculosis* (Mtb)-infected macrophages.**

BMDMs were incubated with 20 μg/ml Mtb LAM, 5 μg/ml Rv3463 or mixture of LAM and Rv3463 for 3 min and then treated with LAM, Rv3463, or medium. The cells loaded with Flou-4/AM were analyzed by confocal microscopy. Scale bar, 10 μm. *p < 0.05, ***p* < 0.01 and ****p* < 0.001 for treatment compared to infection only. *n.s*., no significant difference.


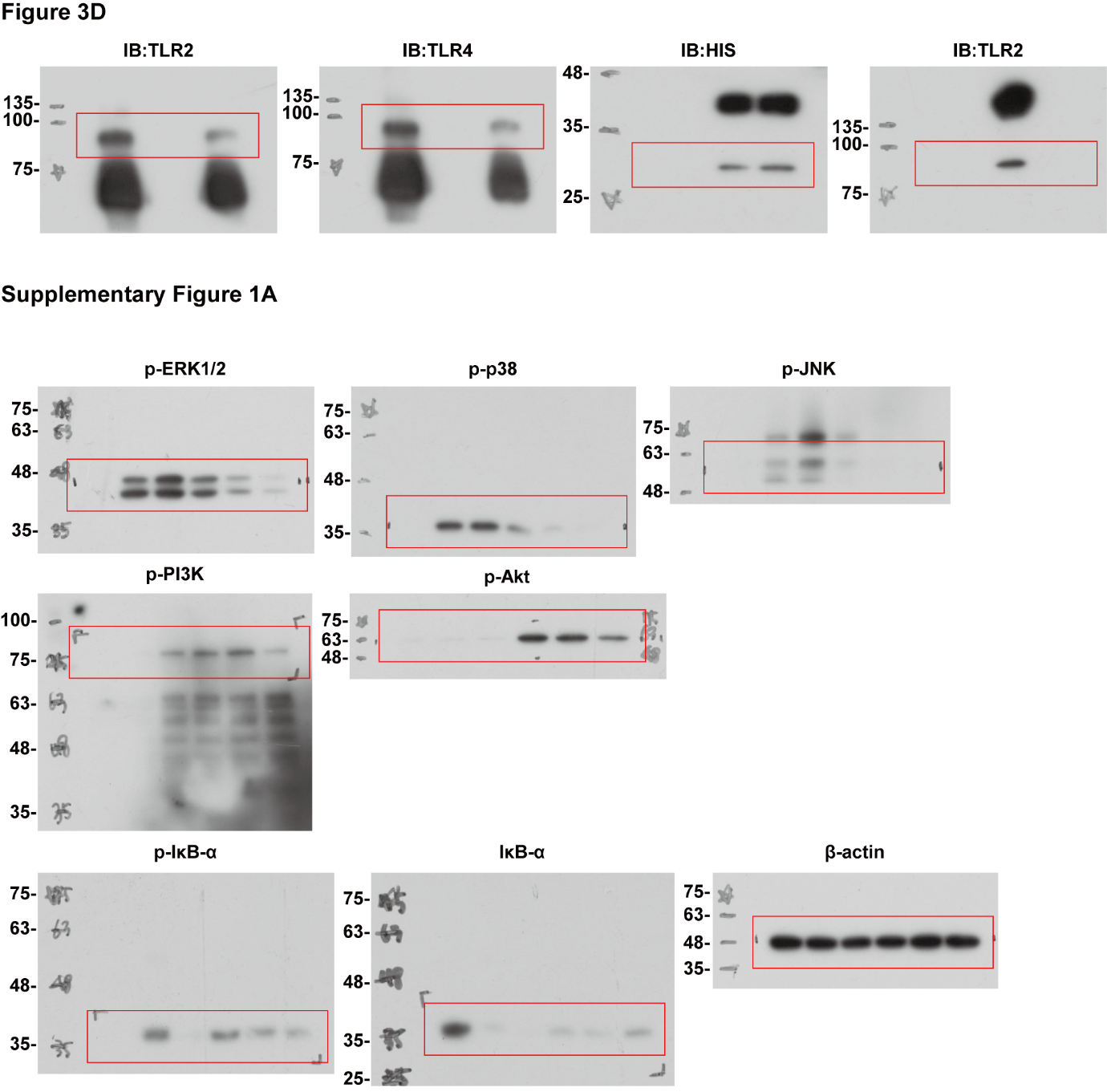


**Supplementary Figure 11.** **Full-length blots in the main paper are presented. Uncropped blots from Figure 3 and Supplementary Figure 1.**

**
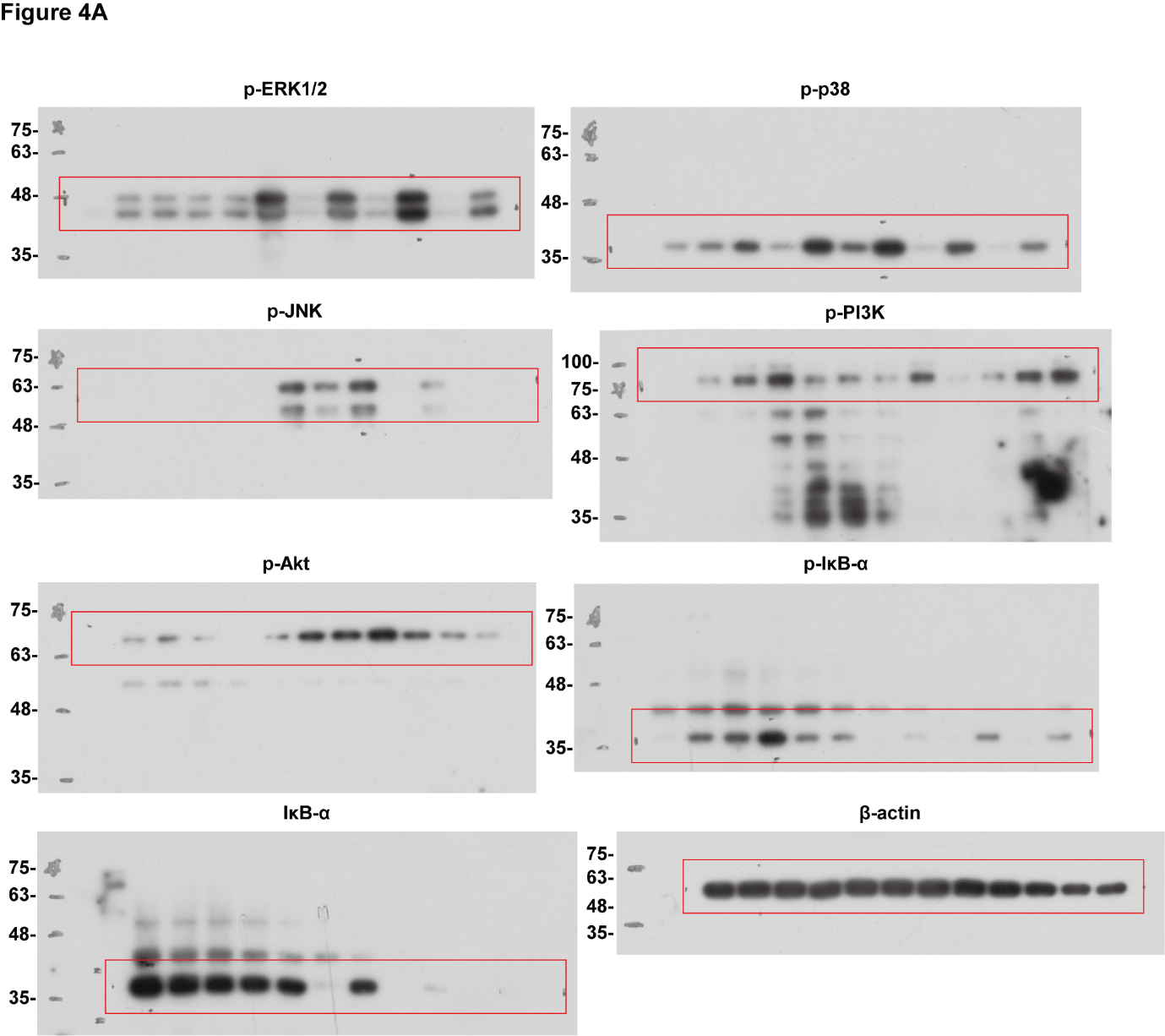
**

**Supplementary Figure 12.** **Full-length blots in the main paper are presented. Uncropped blots from Figure 4.**

**
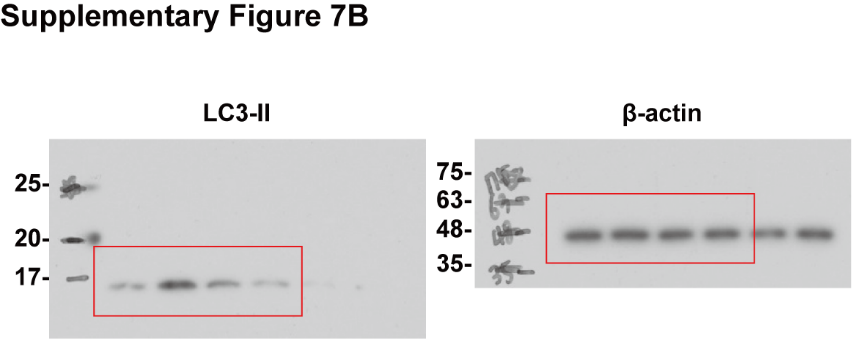
**

**Supplementary Figure 13.** **Full-length blots in the supplementary information are presented. Uncropped blots from Supplementary Figure 7.**

**
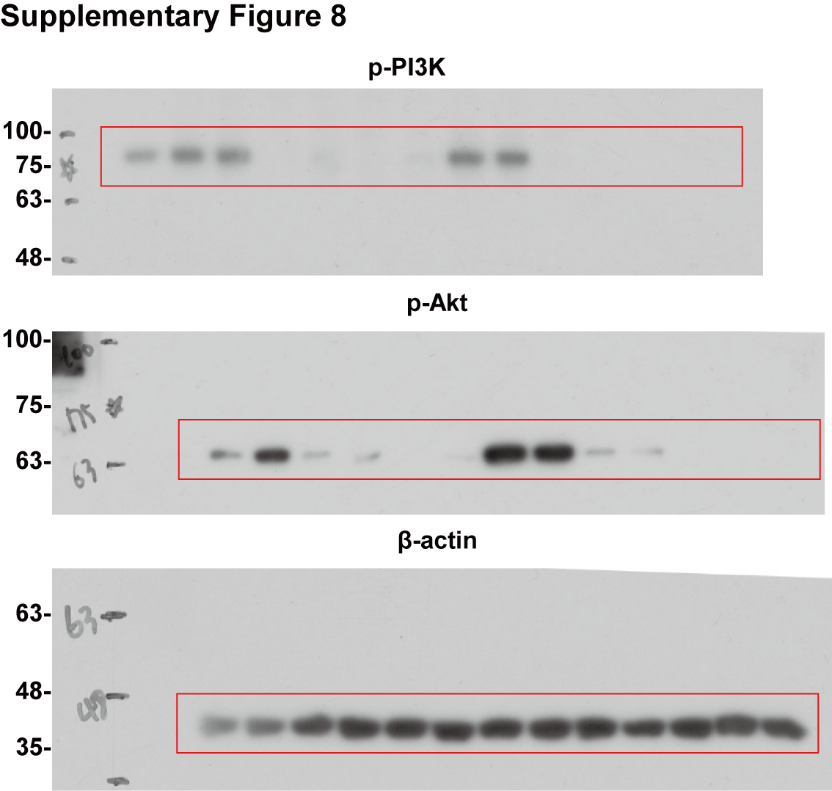
**

**Supplementary Figure 14.** **Full-length blots in the supplementary information are presented. Uncropped blots from Supplementary Figure 8.**
